# Supplementary material for: Nucleoporin93 limits Yap activity to prevent endothelial cell senescence
Source: Aging Cell. 2024 Feb 13;23(4):e14095. doi: 10.1111/acel.14095 (PMC11019141; doi:10.1111/acel.14095)
Supplement: Supplementary file 1 — Figures S1–S6 [file ACEL-23-e14095-s001.zip › acel14095-sup-0001-FiguresS1-S6.pdf]

Figure S1

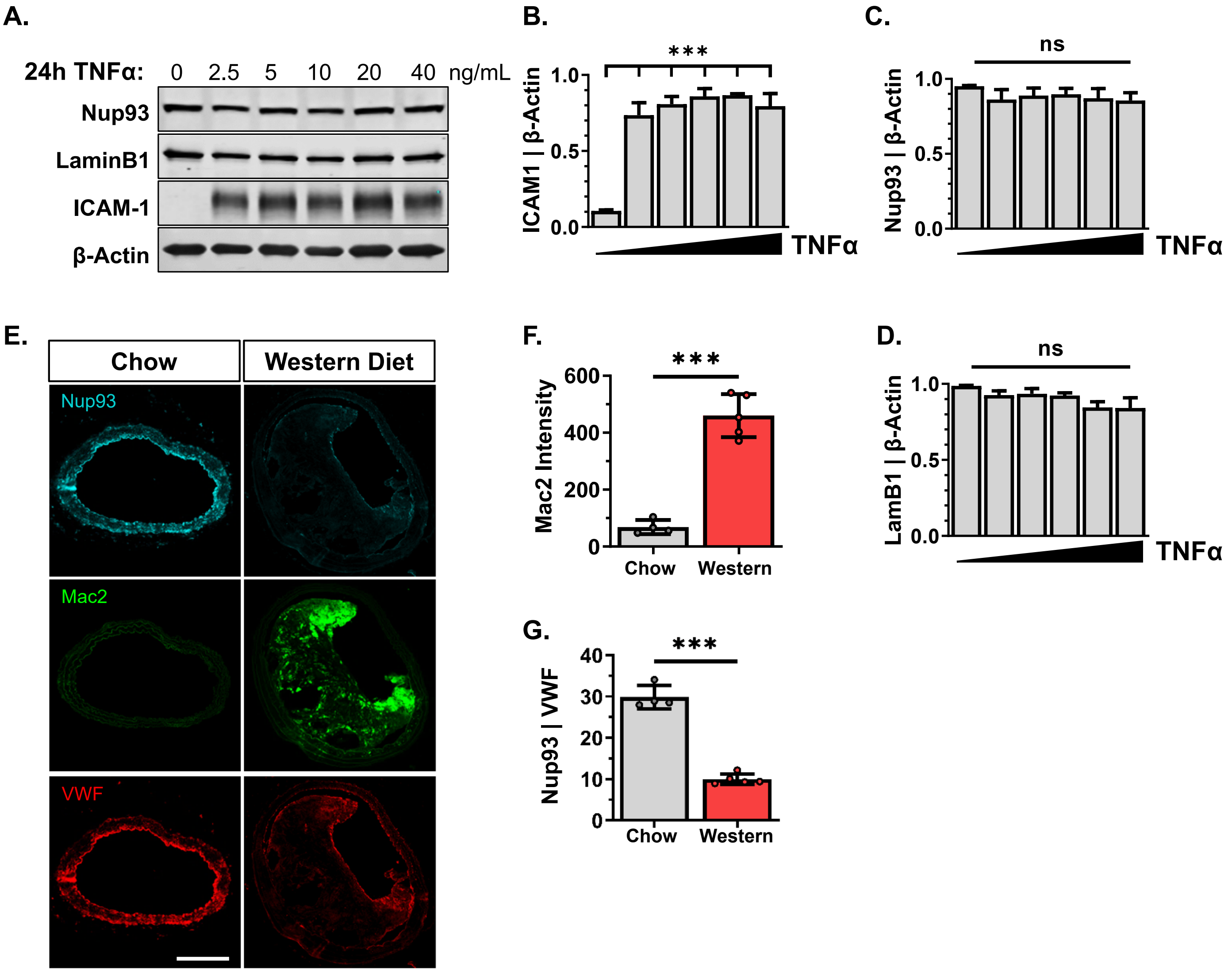
A.
24h TNFα: 0 2.5 5 10 20 40 ng/mL
Nup93
LaminB1
ICAM-1
β-Actin
B.
***
ICAM1 | β-Actin
1.0
0.5
0.0
TNFα
C.
ns
Nup93 | β-Actin
1.0
0.5
0.0
TNFα
E.
Chow
Western Diet
Nup93
Mac2
VWF
F.
***
Mac2 Intensity
600
400
200
0
Chow
Western
D.
ns
LamB1 | β-Actin
1.0
0.5
0.0
TNFα
G.
***
Nup93 | VWF
40
30
20
10
0
Chow
Western

Figure S2

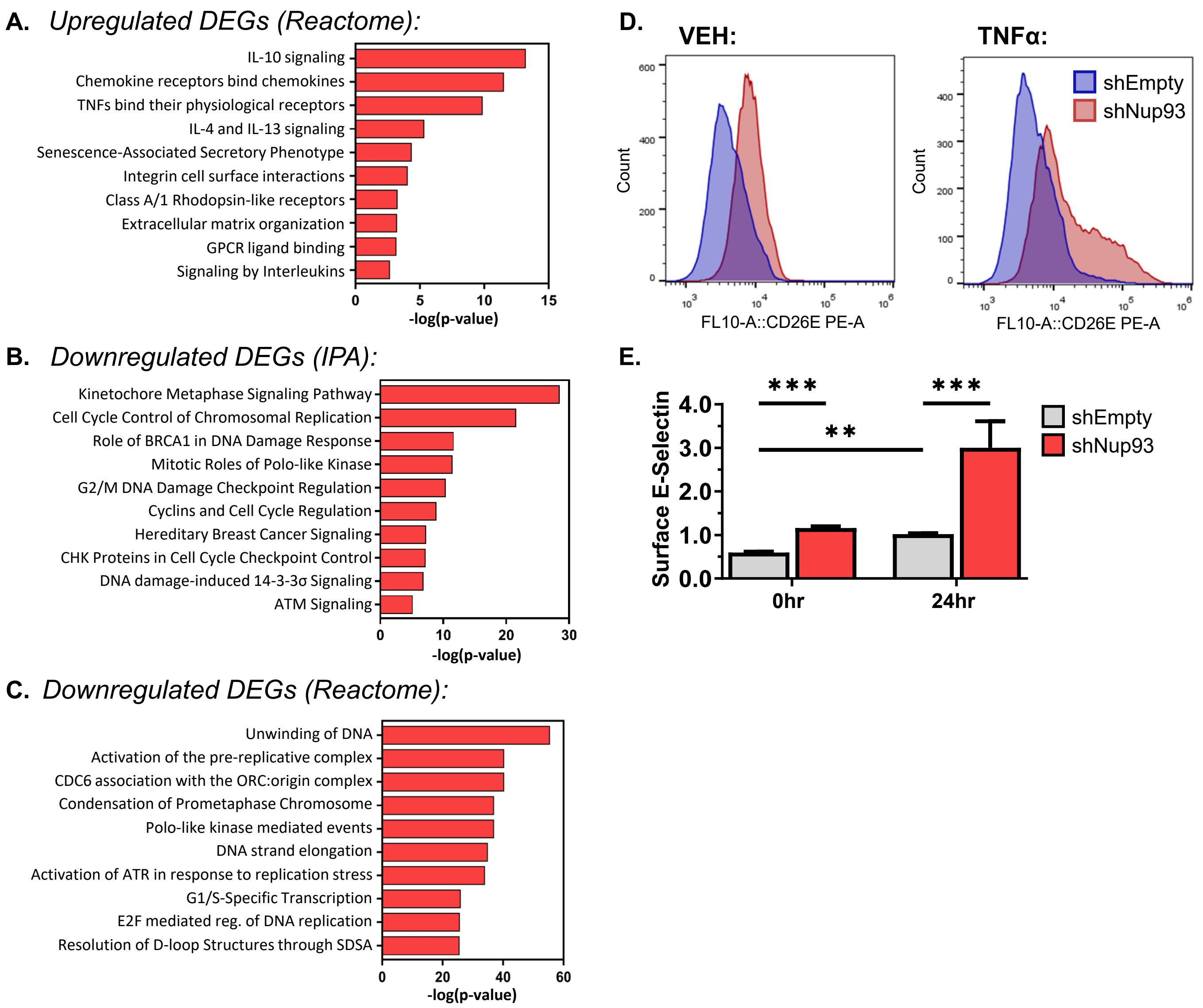
A. Upregulated DEGs (Reactome):
IL-10 signaling
Chemokine receptors bind chemokines
TNFs bind their physiological receptors
IL-4 and IL-13 signaling
Senescence-Associated Secretory Phenotype
Integrin cell surface interactions
Class A/1 Rhodopsin-like receptors
Extracellular matrix organization
GPCR ligand binding
Signaling by Interleukins
0
5
10
15
-log(p-value)
B. Downregulated DEGs (IPA):
Kinetochore Metaphase Signaling Pathway
Cell Cycle Control of Chromosomal Replication
Role of BRCA1 in DNA Damage Response
Mitotic Roles of Polo-like Kinase
G2/M DNA Damage Checkpoint Regulation
Cyclins and Cell Cycle Regulation
Hereditary Breast Cancer Signaling
CHK Proteins in Cell Cycle Checkpoint Control
DNA damage-induced 14-3-3σ Signaling
ATM Signaling
0
10
20
30
-log(p-value)
C. Downregulated DEGs (Reactome):
Unwinding of DNA
Activation of the pre-replicative complex
CDC6 association with the ORC:origin complex
Condensation of Prometaphase Chromosome
Polo-like kinase mediated events
DNA strand elongation
Activation of ATR in response to replication stress
G1/S-Specific Transcription
E2F mediated reg. of DNA replication
Resolution of D-loop Structures through SDSA
0
20
40
60
-log(p-value)
D.
VEH:
TNFα:
shEmpty
shNup93
Count
FL10-A::CD26E PE-A
E.
Surface E-Selectin
4.0
3.0
2.0
1.0
0.0
***
**
***
0hr
24hr
shEmpty
shNup93

Figure S3

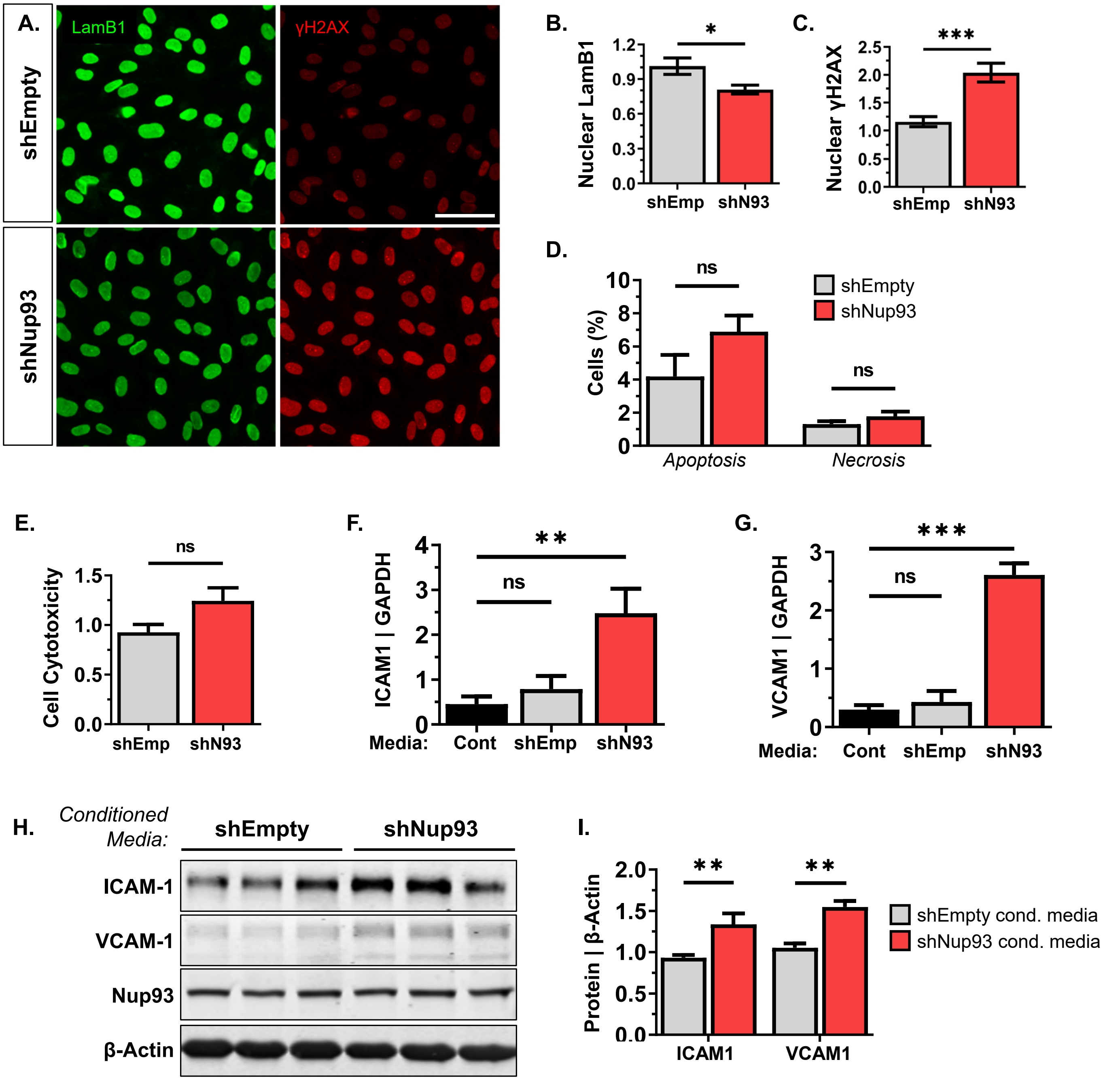

A.
LamB1
γH2AX
shEmpty
shNup93
B.
Nuclear LamB1
*
shEmp
shN93
C.
Nuclear γH2AX
***
shEmp
shN93
D.
Cells (%)
ns
ns
shEmpty
shNup93
Apoptosis
Necrosis
E.
Cell Cytotoxicity
ns
shEmp
shN93
F.
ICAM1 | GAPDH
**
ns
Media:
Cont
shEmp
shN93
G.
VCAM1 | GAPDH
***
ns
Media:
Cont
shEmp
shN93
H.
Conditioned Media:
shEmpty
shNup93
ICAM-1
VCAM-1
Nup93
β-Actin
I.
Protein | β-Actin
**
**
shEmpty cond. media
shNup93 cond. media
ICAM1
VCAM1

Figure S4

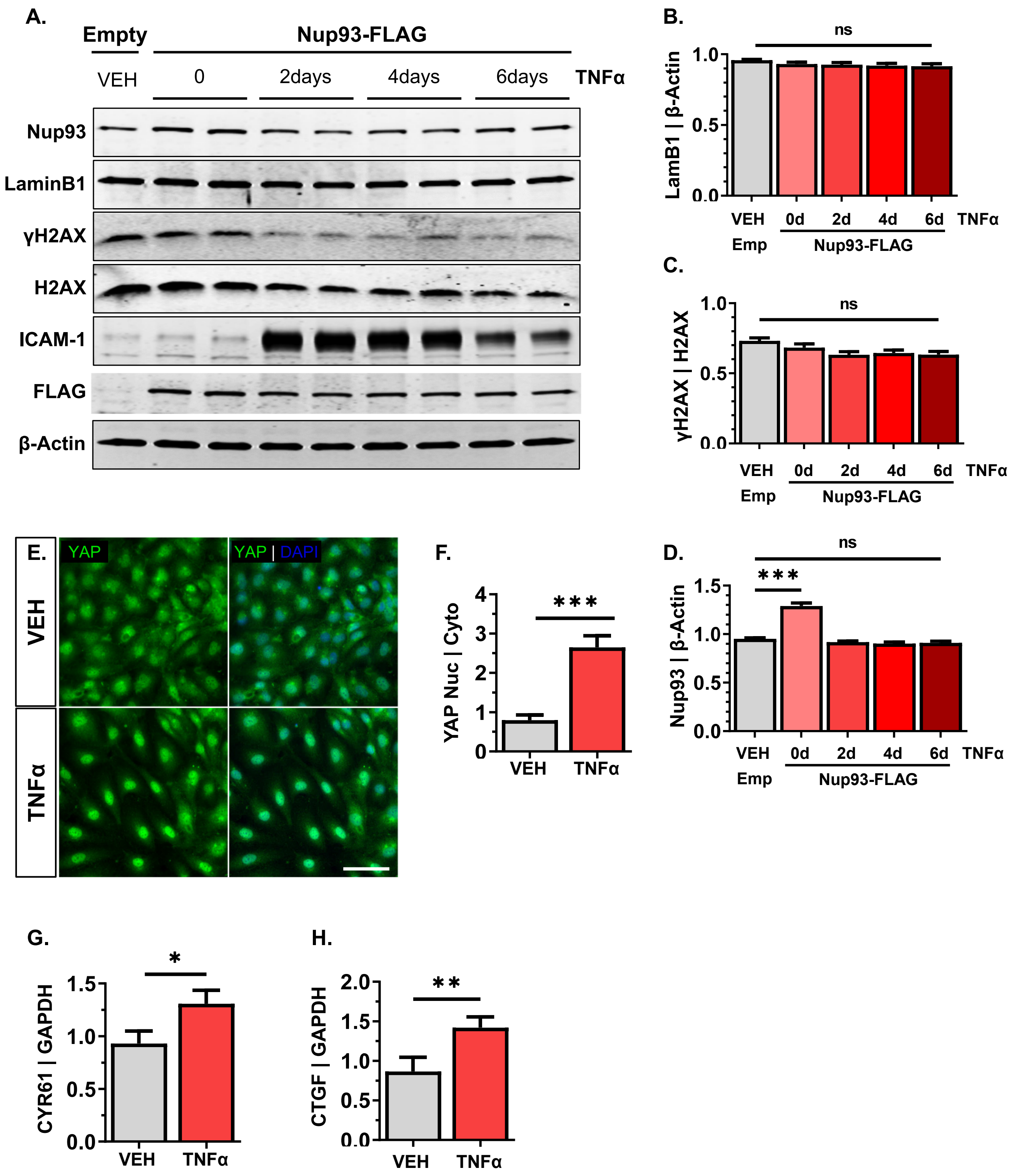
A.
Empty
Nup93-FLAG
VEH
0
2days
4days
6days
TNFα
Nup93
LaminB1
γH2AX
H2AX
ICAM-1
FLAG
β-Actin
B.
ns
LamB1 | β-Actin
1.0
0.5
0.0
VEH
0d
2d
4d
6d
TNFα
Emp
Nup93-FLAG
C.
ns
γH2AX | H2AX
1.0
0.5
0.0
VEH
0d
2d
4d
6d
TNFα
Emp
Nup93-FLAG
E.
YAP
YAP | DAPI
VEH
TNFα
F.
***
YAP Nuc | Cyto
4
3
2
1
0
VEH
TNFα
D.
ns
***
Nup93 | β-Actin
1.5
1.0
0.5
0.0
VEH
0d
2d
4d
6d
TNFα
Emp
Nup93-FLAG
G.
*
CYR61 | GAPDH
1.5
1.0
0.5
0.0
VEH
TNFα
H.
**
CTGF | GAPDH
2.0
1.5
1.0
0.5
0.0
VEH
TNFα

Figure S5

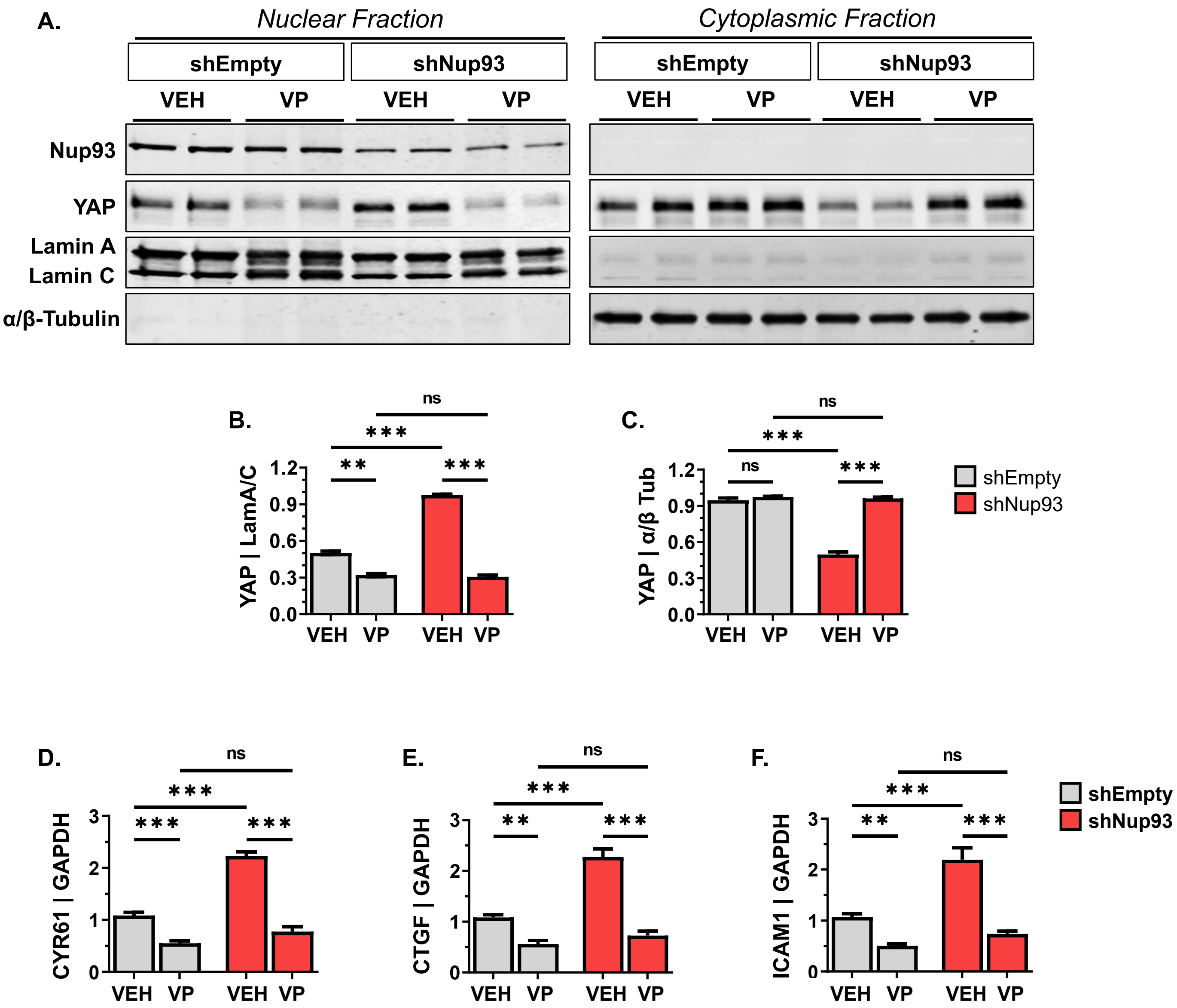
A.
Nuclear Fraction
Cytoplasmic Fraction
shEmpty
shNup93
VEH
VP
Nup93
YAP
Lamin A
Lamin C
α/β-Tubulin
B.
YAP | LamA/C
C.
YAP | α/β Tub
ns
***
**
shEmpty
shNup93
D.
CYR61 | GAPDH
E.
CTGF | GAPDH
F.
ICAM1 | GAPDH

Figure S6

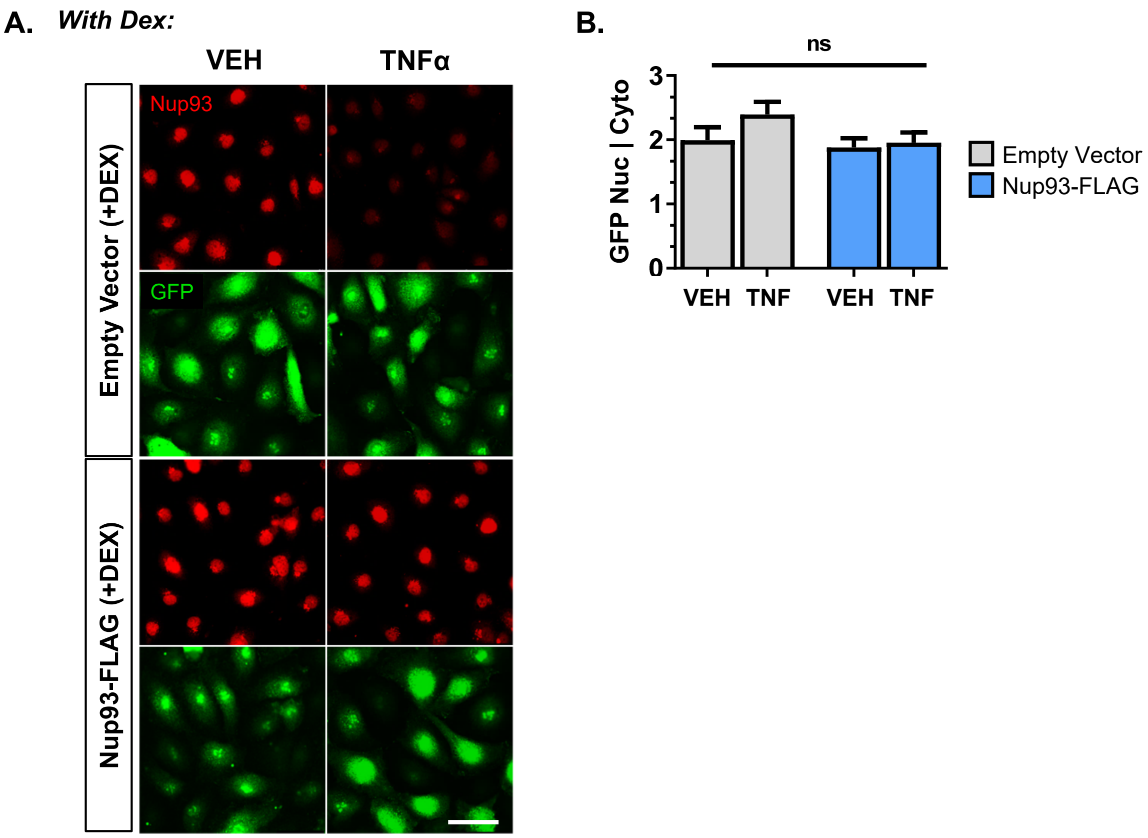
A.
With Dex:
VEH
TNFα
Empty Vector (+DEX)
Nup93-FLAG (+DEX)
Nup93
GFP
B.
ns
GFP Nuc | Cyto
3
2
1
0
VEH
TNF
VEH
TNF
Empty Vector
Nup93-FLAG
