# Supplementary material for: Nucleoporin93 limits Yap activity to prevent endothelial cell senescence
Source: Aging Cell. 2024 Feb 13;23(4):e14095. doi: 10.1111/acel.14095 (PMC11019141; doi:10.1111/acel.14095)
Supplement: Supplementary file 1 — Figures S1–S6 [file ACEL-23-e14095-s001.zip › FiguresS1-S6_Legends.docx]

**Figure S1. Acute inflammation conditions do not affect endothelial Nup93 protein levels. (A)**. Acute exposure to TNFα (24hrs) over a range of concentrations (2.5-40ng/mL) significantly increases ICAM1 expression without any effect on Nup93 or LaminB1 expression (n=3). Quantified in **(B-D)**. **(E)** ApoE-null mice were challenged with a 12-week Western Diet (or maintained on standard chow diet) for atherosclerotic plaque visualization. Immunofluorescence staining of the brachiocephalic artery (BCA) indicates the presence of atherosclerotic lesion formation and increased macrophage infiltration, as assessed through Mac2 staining. Endothelial Nup93 expression declines under Western Diet conditions, as determined by Nup93 intensity in VWF-positive regions (n=4 Chow, n=5 Western Diet). Quantified in **(F)** and **(G)** in fluorescence intensity units. **** p<0.001*

**Figure S2. Endothelial loss of Nup93 promotes a genetic signature indicative of cellular senescence. (A)** Pathway analyses (Reactome) of the upregulated DEGs identifies several inflammation-associated pathways, including the senescence-associated secretory phenotype. Significantly downregulated genes with loss of Nup93 were also subjected to pathways analyses using both **(B)** IPA and **(C)** Reactome platforms. Common pathways include those associated with cell cycle regulation and the DNA damage response. **(D)** FACS plots of surface E-selectin show significantly higher levels in Nup93 knockdown HRECs. Quantified in **(E)**. n=3, **** p<0.001, ** p<0.01*

**Figure S3. Loss of Nup93 leads to endothelial senescence and acquisition of SASP. (A)** Loss of Nup93 in primary HRECs leads to a visible change in both LaminB1 and γH2AX levels to indicate cellular senescence. Quantified in **(B)** and **(C)**. **(D)** FACS analysis of HRECs (shEmpty & shNup93) stained with PI and AnnexinV to quantify apoptosis and necrosis indicate no obvious differences. **(E)** No significant effects on cell cytotoxicity in shNup93-transduced HRECs 72hours post-infection. **(F&G)** RT-qPCR analysis and **(H)** immunoblotting of primary HRECs treated with conditioned media from the indicated groups. Representative immunoblot shows technical triplicates and quantified in **(I)**. Scale bar=50µm. *n=3, *** p<0.001, ** p<0.01,* p<0.05*

**Figure S4. Chronic inflammation in ECs triggers Yap signaling where features of senescence are prevented by moderate overexpression of Nup93. (A)** Nup93 overexpression in primary HRECs prior to chronic inflammation has no effect on LaminB1 or γH2AX levels. Quantified in **(B)** and **(C)**. **(E&F)** Chronic inflammation (TNFα [10ng/mL]; 6 days) in primary HRECs leads to a significant increase in nuclear Yap accumulation and **(G&H)** downstream target gene expression. Scale bar=50µm. *n=3, *** p<0.001, ** p<0.01, * p<0.05*

**Figure S5. Verteporfin treatment prevents Yap nuclear localization in both control and Nup93-depleted primary HRECs. (A)** HRECs (shEmpty & shNup93) were treated with verteportin (VP [0.5µM], 24hrs) prior to fractionation and subsequent immunoblotting of Yap in nuclear and cytoplasmic fractions. Quantification of Yap in the nucleus **(B)** and **(C)** cytoplasm. **(DF)** RT-qPCR analysis indicates a significant reduction in Yap target gene expression upon VP treatment in both groups. *n=3, *** p<0.001, ** p<0.01*

**Figure S6. Restoring Nup93 expression in already senescent ECs does not affect steroidinduced nuclear localization of the RGG construct.** HRECs were transduced with the RGG construct using lentiviral methods. Transduced cells were then exposed to the chronic inflammation model followed by lentiviral-mediated expression of exogenous Nup93. **(A)** Exogenous expression of Nup93 does not affect the performance of the RGG construct, as treatment with dexamethasone (DEX [1µM], 30 minutes) induces nuclear GFP signal across all conditions. GFP nuclear-to-cytoplasmic intensities quantified in **(B)**. Scale bar=50µm. n=3.
