# Supplementary material for: Nucleoporin93 limits Yap activity to prevent endothelial cell senescence
Source: Aging Cell. 2024 Feb 13;23(4):e14095. doi: 10.1111/acel.14095 (PMC11019141; doi:10.1111/acel.14095)
Supplement: Supplementary file 2 — Tables S1–S2 [file ACEL-23-e14095-s002.pdf]

Table S1. RT-qPCR primers for detection of human transcripts

Target gene	Forward sequence (5'→3')	Reverse sequence (5'→3')	Product Size (bp)
CYR61	TGAAGCGGCTCCCTGTTTT	CGGGTTTCTTTCACAAGGCG	175
CTGF	ACCGACTGGAAGACACGTTTG	CCAGGTCAGCTTCGCAAGG	192
ICAM1	TTGGGCATAGAGACCCCGTT	GCACATTGCTCAGTTCATACACC	93
VCAM1	CAGTAAGGCAGGCTGTAAAAGA	TGGAGCTGGTAGACCCTCG	144
GAPDH	CTCTCTGCTCCTCCTGTTCGAC	TGAGCGATGTGGCTCGGCT	71

Table S2. Differentially expressed genes in shNup93-transduced primary HRECs (compared to shEmpty control).

Gene_ID	shNup93 A	shNup93 B	shNup93 C	shEmpty A	shEmpty B	shEmpty C	log2FoldChange	padj	GeneName
ENSG00000114812	22.32649827	13.55212335	28.12189076	0	0	0	6.936330717	1.91E-06	VIPR1
ENSG00000137203	23.3413391	9.382239244	16.22416775	0	0	0	6.551147929	1.77E-05	TFAP2A
ENSG00000183160	19.28197578	8.339768217	19.4690013	0	0	0	6.494275047	2.39E-05	TMEM119
ENSG00000179399	56.83108651	40.65637006	40.01961377	0	0	2.000122788	6.179650147	2.12E-07	GPC5
ENSG00000183090	15.22261246	5.212355136	11.89772301	0	0	0	5.952561525	0.00035493	FREM3
ENSG00000015520	14.20777163	23.97683362	15.14255656	0.879212552	0	0	5.712135973	0.000160985	NPC1L1
ENSG00000006210	155.2706471	115.714284	122.2220637	1.758425104	2.892657035	3.000184182	5.699468603	3.60E-22	CX3CL1
ENSG00000163606	2.029681661	8.339768217	16.22416775	0	0	0	5.666727829	0.001742966	CD200R1
ENSG00000215914	3.044522491	9.382239244	11.89772301	0	0	0	5.538978806	0.002212355	MMP23A
ENSG00000130294	10.1484083	8.339768217	5.408055916	0	0	0	5.517327128	0.001966916	KIF1A
ENSG00000233930	7.103885813	13.55212335	2.163222366	0	0	0	5.451012743	0.003601496	KRTAP5-AS1
ENSG00000043462	9.133567474	10.42471027	23.79544603	0.879212552	0	0	5.410841986	0.000674412	LCP2
ENSG00000198771	5.074204152	9.382239244	7.571278282	0	0	0	5.397630586	0.003130131	RCSD1
ENSG00000163121	8.118726644	3.127413081	9.734500648	0	0	0	5.327327452	0.004581153	NEURL3
ENSG00000224294	9.133567474	7.29729719	4.326444732	0	0	0	5.314402137	0.004395954	PINCR
ENSG00000104490	10.1484083	9.382239244	1.081611183	0	0	0	5.305471582	0.006816555	NCALD
ENSG00000070985	11.16324914	8.339768217	1.081611183	0	0	0	5.303788972	0.006918151	TRPM5
ENSG00000183114	9.133567474	1.042471027	9.734500648	0	0	0	5.252078773	0.008075344	FAM43B
ENSG00000038945	8.118726644	7.29729719	4.326444732	0	0	0	5.241824996	0.005619906	MSR1
ENSG00000132517	2.029681661	10.42471027	6.489667099	0	0	0	5.179416518	0.008566015	SLC52A1
ENSG00000007314	15.22261246	5.212355136	16.22416775	0.879212552	0	0	5.170990293	0.001717142	SCN4A
ENSG00000137491	45.66783737	51.08108033	59.48861507	0.879212552	3.856876047	0	5.045747142	3.34E-10	SLCO2B1
ENSG00000249464	20.29681661	16.67953643	22.71383485	0.879212552	0	1.000061394	4.981906613	0.000183171	LINC01091
ENSG00000166592	47.69751903	51.08108033	46.50928087	1.758425104	0	3.000184182	4.94115196	8.99E-10	RRAD
ENSG00000135253	12.17808997	5.212355136	11.89772301	0.879212552	0	0	4.847441106	0.004943397	KCP
ENSG00000074317	13.1929308	19.80694952	18.38739011	1.758425104	0	0	4.781150748	0.000572388	SNCB
ENSG00000115607	49.72720069	53.16602238	51.91733679	2.637637656	0.964219012	2.000122788	4.77368359	1.30E-10	IL18RAP
ENSG00000261594	89.30599308	63.59073266	97.34500648	3.516850207	1.928438024	4.000245576	4.726672573	3.14E-16	TPBGL
ENSG00000110324	17.25229412	7.29729719	14.06094538	0.879212552	0	1.000061394	4.354896239	0.003217505	IL10RA
ENSG00000171724	91.33567474	43.78378314	76.794394	2.637637656	2.892657035	5.00030697	4.345207292	2.72E-13	VAT1L
ENSG00000261239	64.94981315	40.65637006	43.26444732	5.275275311	0	2.000122788	4.313820405	1.07E-09	ANKRD26P1
ENSG00000175899	26.38586159	20.84942054	8.652889465	0.879212552	0.964219012	1.000061394	4.302506072	0.000318242	A2M
ENSG00000110848	38.56395156	38.571428	52.99894797	0.879212552	4.821095059	1.000061394	4.292655722	7.64E-09	CD69
ENSG00000107562	47.69751903	47.95366725	88.69211701	3.516850207	0	6.000368364	4.281967078	2.83E-11	CXCL12
ENSG00000162981	32.47490658	23.97683362	17.30577893	0	0	4.000245576	4.267315558	3.73E-05	FAM84A
ENSG00000112299	14.20777163	8.339768217	11.89772301	1.758425104	0	0	4.211324293	0.006085323	VNN1
ENSG00000268894	36.5342699	37.52895698	25.95866839	4.396062759	0	1.000061394	4.159226366	7.51E-07	PLCE1-AS1
ENSG00000164440	19.28197578	10.42471027	19.4690013	1.758425104	0.964219012	0	4.129718607	0.000742908	TXLNB
ENSG00000196208	7.103885813	17.72200746	7.571278282	1.758425104	0	0	4.125043731	0.009394121	GREB1
ENSG00000049249	35.51942907	23.97683362	19.4690013	1.758425104	1.928438024	1.000061394	4.067092153	1.00E-05	TNFRSF9
ENSG00000173110	146.1370796	101.1196896	187.1187347	5.275275311	9.642190118	14.00085951	3.92718638	6.30E-24	HSPA6
ENSG00000267640	23.3413391	26.06177568	7.571278282	1.758425104	0	2.000122788	3.918606337	0.000453654	AC016582.3
ENSG00000105246	76.11306229	75.05791395	73.54956045	6.154487863	3.856876047	5.00030697	3.896272971	4.01E-15	EBI3
ENSG00000162643	9.133567474	18.76447849	14.06094538	0	0	3.000184182	3.863129364	0.003058912	WDR63
ENSG00000168453	20.29681661	8.339768217	11.89772301	0.879212552	0	2.000122788	3.833205273	0.003176896	HR
ENSG00000170323	539.8953218	463.8996071	468.3376423	41.32298994	34.71188443	34.00208739	3.737316218	3.32E-88	FABP4
ENSG00000080709	19.28197578	20.84942054	20.55061248	1.758425104	1.928438024	1.000061394	3.68570734	0.000145561	KCNN2
ENSG00000107105	16.23745329	10.42471027	9.734500648	0	0.964219012	2.000122788	3.664944733	0.005641466	ELAVL2
ENSG00000143869	1138.651412	938.2239244	883.6763366	86.16283008	76.17330193	73.00448176	3.650096433	5.09E-146	GDF7
ENSG00000007908	981.3510831	757.8764367	949.6546188	79.12912967	85.81549205	67.00411339	3.533155639	1.38E-128	SELE
ENSG00000206337	40.59363322	25.01930465	27.04027958	4.396062759	2.892657035	1.000061394	3.458453072	4.73E-06	HCP5
ENSG00000181634	2041.859751	1737.799202	1818.188399	193.4267614	150.4181658	173.0106211	3.4342239	5.10E-250	TNFSF15
ENSG00000064300	67.99433564	69.84555882	63.8150598	7.912912967	4.821095059	6.000368364	3.419564745	9.58E-13	NGFR
ENSG00000102109	12.17808997	29.18918876	8.652889465	0.879212552	0	4.000245576	3.387194711	0.002692348	PCSK1N
ENSG00000154928	151.2112837	148.0308859	142.7726762	15.82582593	15.42750419	13.00079812	3.316758888	1.22E-26	EPHB1
ENSG00000171246	27.40070242	9.382239244	29.20350194	1.758425104	0.964219012	4.000245576	3.30925515	0.000361463	NPTX1
ENSG00000004468	17.25229412	12.50965233	7.571278282	0.879212552	2.892657035	0	3.306634156	0.008218277	CD38
ENSG00000113645	12.17808997	15.63706541	8.652889465	1.758425104	1.928438024	0	3.282945308	0.008018466	WWC1
ENSG00000186827	18.26713495	21.89189157	23.79544603	2.637637656	1.928438024	2.000122788	3.27588472	0.000180361	TNFRSF4
ENSG00000105825	1109.221028	1084.169868	1087.019239	116.0560568	104.1356533	123.0075515	3.257233846	5.82E-181	TFPI2
ENSG00000160223	32.47490658	57.33590649	35.69316904	2.637637656	3.856876047	7.000429757	3.235179789	2.31E-07	ICOSLG
ENSG00000157404	375.4911073	453.4748968	334.2178556	39.56456483	36.64032245	48.00294691	3.230405395	5.77E-58	KIT
ENSG00000163735	347.075564	265.8301119	338.5443003	37.80613973	28.92657035	37.00227158	3.195425936	5.81E-49	CXCL5
ENSG00000019582	53.78656402	63.59073266	43.26444732	5.275275311	2.892657035	10.00061394	3.155650159	4.46E-09	CD74
ENSG00000056558	519.5985052	527.4903397	587.3148724	63.30330373	57.85314071	68.00417479	3.111473758	1.08E-86	TRAF1
ENSG00000007129	45.66783737	34.4015439	25.95866839	3.516850207	3.856876047	5.00030697	3.107196203	2.10E-06	CEACAM21
ENSG00000069122	905.2380208	694.2857041	726.842715	95.83416815	69.42376885	105.0064464	3.106043861	8.06E-92	ADGRF5
ENSG00000141668	69.00917647	71.93050087	85.44728347	12.30897573	8.677971106	5.00030697	3.105880214	1.19E-12	CBLN2
ENSG00000107719	587.5928408	513.9382164	522.4182014	65.94094139	66.53111182	58.00356085	3.090130572	4.61E-85	PALD1
ENSG00000102760	594.6967267	581.6988331	612.1919296	70.33700415	63.63845478	81.00497291	3.058381823	1.40E-94	RGCC
ENSG00000205710	27.40070242	21.89189157	28.12189076	2.637637656	4.821095059	2.000122788	3.033319267	6.88E-05	C17orf107
ENSG00000237988	55.81624568	74.01544293	60.57022625	12.30897573	2.892657035	8.000491151	3.021345845	5.60E-10	OR2I1P
ENSG00000174059	937.7129274	872.5482497	879.3498919	124.8481824	86.77971106	122.0074901	3.009093784	3.32E-122	CD34
ENSG00000162692	1244.194858	1078.957513	1263.321862	146.8284962	127.2769096	175.0107439	2.999300029	8.13E-146	VCAM1
ENSG00000163083	25.37102076	30.23165979	19.4690013	1.758425104	3.856876047	4.000245576	2.981089304	0.000114807	INHBB
ENSG00000137285	14.20777163	3.127413081	42.18283614	0.879212552	2.892657035	4.000245576	2.953065456	0.005748285	TUBB2B
ENSG00000115008	51.75688235	25.01930465	29.20350194	4.396062759	5.785314071	4.000245576	2.903537904	8.16E-06	IL1A
ENSG00000120337	667.7562665	486.8339697	634.9057645	99.35101836	69.42376885	70.00429757	2.899674803	1.06E-65	TNFSF18
ENSG00000142235	55.81624568	37.52895698	87.61050583	6.154487863	9.642190118	9.000552545	2.873084265	1.37E-08	LMTK3
ENSG00000185607	8.118726644	33.35907287	25.95866839	1.758425104	4.821095059	3.000184182	2.824074691	0.001231733	ACTBP7
ENSG00000135477	20.29681661	11.4671813	15.14255656	0.879212552	5.785314071	0	2.821086118	0.008374621	KRT87P
ENSG00000108932	62.92013149	108.4169868	85.44728347	11.42976317	17.35594221	8.000491151	2.801109597	8.05E-12	SLC16A6
ENSG00000121966	181.6565087	161.5830092	169.8129557	27.25558911	26.99813233	20.00122788	2.784032409	9.76E-26	CXCR4
ENSG00000108691	3892.929426	3586.100333	3582.296238	538.9572943	514.8929523	559.0343192	2.77824421	0	CCL2
ENSG00000137726	29.43038408	21.89189157	30.28511313	5.275275311	4.821095059	2.000122788	2.740610247	0.00012197	FXYD6
ENSG00000110876	58.86076817	65.67567471	49.75411442	14.94661338	3.856876047	7.000429757	2.736780041	3.59E-08	SELPLG
ENSG00000136002	13.1929308	17.72200746	19.4690013	1.758425104	2.892657035	3.000184182	2.728329109	0.003167921	ARHGEF4
ENSG00000274615	127.8699446	119.8841681	67.05989335	20.22188869	19.28438024	8.000491151	2.719464136	2.90E-12	AC233968.1
ENSG00000232480	32.47490658	42.74131211	62.73344862	5.275275311	4.821095059	11.00067533	2.718825596	1.20E-06	TGFB2-AS1
ENSG00000166670	70.0240173	59.42084855	70.3047269	8.792125519	7.713752095	14.00085951	2.71835041	3.83E-10	MMP10

ENSG00000189056	364.3278581	277.2972932	297.4430754	36.04771463	36.64032245	71.00435897	2.717360675	2.44E-32	RELN
ENSG00000140323	87.27631142	95.9073345	108.1611183	12.30897573	16.3917232	16.0009823	2.709021228	1.49E-14	DISP2
ENSG00000162490	66.97949481	61.5057906	81.12083873	12.30897573	7.713752095	13.00079812	2.664244286	2.57E-10	DRAXIN
ENSG00000124257	27.40070242	31.27413081	31.36672431	4.396062759	3.856876047	6.000368364	2.663630996	4.83E-05	NEURL2
ENSG00000198576	5.074204152	25.01930465	29.20350194	5.275275311	0	4.000245576	2.661955491	0.007968275	ARC
ENSG00000182985	208.0423702	183.4749008	160.0784551	29.01401421	36.64032245	22.00135067	2.652204881	3.32E-24	CADM1
ENSG00000048052	88.29115225	70.88802985	100.58984	6.154487863	15.42750419	21.00128927	2.625589709	9.01E-11	HDAC9
ENSG00000271978	47.69751903	14.59459438	11.89772301	3.516850207	0.964219012	8.000491151	2.584623286	0.003215674	AL359643.2
ENSG00000186472	20.29681661	13.55212335	11.89772301	0.879212552	3.856876047	3.000184182	2.583889431	0.008619268	PCLO
ENSG00000110042	42.62331488	44.82625417	46.50928087	7.912912967	6.749533083	8.000491151	2.562469825	6.77E-07	DTX4
ENSG00000167642	189.7752353	134.4787625	171.9761781	18.46346359	35.67610344	31.00190321	2.5518225	2.28E-19	SPINT2
ENSG00000135678	224.2798235	124.0540522	155.7520104	34.28928952	21.21281826	31.00190321	2.540386035	1.19E-17	CPM
ENSG00000184524	146.1370796	158.4555961	191.4451794	28.13480166	29.89078937	30.00184182	2.494816789	1.79E-21	CEND1
ENSG00000105499	835.2140035	797.4903358	879.3498919	150.3453464	148.4897278	147.0090249	2.49370082	3.75E-105	PLA2G4C
ENSG00000109072	28.41554325	26.06177568	34.61155786	7.033700415	3.856876047	5.00030697	2.477581862	0.000136476	VTN
ENSG00000137809	32.47490658	12.50965233	17.30577893	2.637637656	6.749533083	2.000122788	2.456021918	0.003993506	ITGA11
ENSG00000023445	716.4776263	678.6486387	706.2921026	145.9492836	133.0622236	108.0066305	2.436548076	6.68E-80	BIRC3
ENSG00000172575	20.29681661	9.382239244	20.55061248	5.275275311	1.928438024	2.000122788	2.427978079	0.009392124	RASGRP1
ENSG00000145911	397.8176055	333.5907287	359.0949128	70.33700415	74.24486391	58.00356085	2.426407813	2.58E-43	N4BP3
ENSG00000146216	20.29681661	23.97683362	16.22416775	4.396062759	5.785314071	1.000061394	2.425373413	0.00343312	TTBK1
ENSG00000013293	31.46006574	33.35907287	29.20350194	4.396062759	5.785314071	8.000491151	2.380672204	0.000119613	SLC7A14
ENSG00000272636	75.09822146	84.4401532	65.97828217	12.30897573	25.06969431	6.000368364	2.376798013	4.25E-08	DOC2B
ENSG00000105605	197.8939619	176.1776036	187.1187347	29.89322676	35.67610344	43.00263994	2.375873612	4.78E-23	CACNG7
ENSG00000108551	78.14274395	81.31274012	115.7323966	23.7387389	14.46328518	15.00092091	2.360858958	1.01E-10	RASD1
ENSG00000162772	453.6338512	526.4478687	427.2364173	95.83416815	103.1714343	77.00472733	2.348112313	3.91E-49	ATF3
ENSG00000106069	13.1929308	15.63706541	23.79544603	4.396062759	0.964219012	5.00030697	2.340603669	0.009095381	CHN2
ENSG00000163734	275.0218651	237.6833942	232.5464044	49.2359029	44.35407454	55.00337667	2.327993124	2.43E-29	CXCL3
ENSG00000110900	281.11091	404.4787585	328.8097997	82.64597988	58.81735972	62.00380642	2.312708786	1.97E-31	TSPAN11
ENSG00000198889	64.94981315	57.33590649	62.73344862	17.58425104	5.785314071	14.00085951	2.299785366	2.42E-07	DCAF12L1
ENSG00000137501	20.29681661	14.59459438	16.22416775	3.516850207	1.928438024	5.00030697	2.295428939	0.008810091	SYTL2
ENSG00000028277	199.9236436	203.2818503	158.9968439	45.7190527	36.64032245	32.00196461	2.292347651	7.03E-21	POU2F2
ENSG00000114654	42.62331488	42.74131211	42.18283614	6.154487863	15.42750419	5.00030697	2.265447167	3.21E-05	EFCC1
ENSG00000182752	140.0480346	116.756755	84.36567228	29.01401421	21.21281826	21.00128927	2.255674183	4.34E-12	PAPPA
ENSG00000185101	51.75688235	39.61389903	61.65183744	12.30897573	8.677971106	11.00067533	2.254188968	2.05E-06	ANO9
ENSG00000259081	26.38586159	17.72200746	19.4690013	2.637637656	4.821095059	6.000368364	2.253704306	0.003528538	AF111169.3
ENSG00000227507	107.573128	102.1621607	77.87600518	17.58425104	15.42750419	28.00171903	2.243982133	1.23E-10	LTB
ENSG00000198691	83.2169481	53.16602238	62.73344862	20.22188869	5.785314071	16.0009823	2.237889149	6.80E-07	ABCA4
ENSG00000267551	26.38586159	22.9343626	17.30577893	3.516850207	8.677971106	2.000122788	2.232532075	0.004203493	AC005264.1
ENSG00000160013	67.99433564	75.05791395	86.52889465	14.06740083	14.46328518	21.00128927	2.217669012	3.94E-09	PTGIR
ENSG00000188338	32.47490658	39.61389903	37.85639141	5.275275311	11.57062814	7.000429757	2.211174281	9.30E-05	SLC38A3
ENSG00000081041	1336.545374	1052.895737	1470.991209	272.5558911	273.8381994	292.017927	2.203426059	3.69E-82	CXCL2
ENSG00000144339	43.63815571	35.44401492	31.36672431	0.879212552	15.42750419	8.000491151	2.200736121	0.000859186	TMEFF2
ENSG00000065609	27.40070242	22.9343626	18.38739011	6.154487863	6.749533083	2.000122788	2.195616565	0.003282623	SNAP91
ENSG00000118407	105.5434464	120.9266391	105.9978959	25.497164	21.21281826	26.00159624	2.192452406	4.66E-13	FILIP1
ENSG00000163751	52.77172318	56.29343547	67.05989335	15.82582593	12.53484715	10.00061394	2.190676721	3.82E-07	CPA3
ENSG00000100156	91.33567474	112.5868709	75.71278282	21.10110124	14.46328518	26.00159624	2.185556614	5.28E-10	SLC16A8
ENSG00000236609	27.40070242	19.80694952	33.52994668	7.033700415	1.928438024	9.000552545	2.167991779	0.001997201	ZNF853
ENSG00000128298	55.81624568	26.06177568	55.16217034	7.033700415	7.713752095	16.0009823	2.166437283	7.62E-05	BAIAP2L2
ENSG00000008517	2167.700014	2272.586839	2281.117985	503.7887922	536.1057706	509.0312495	2.117738052	1.05E-205	IL32
ENSG00000174672	38.56395156	27.10424671	37.85639141	4.396062759	8.677971106	11.00067533	2.117478469	0.000316511	BRSK2
ENSG00000118946	167.448737	132.3938204	154.6703992	32.53086442	42.42563652	31.00190321	2.101671587	9.78E-16	PCDH17
ENSG00000124749	11.16324914	26.06177568	34.61155786	8.792125519	4.821095059	3.000184182	2.094709238	0.006969495	COL21A1
ENSG00000011201	31.46006574	31.27413081	25.95866839	7.033700415	5.785314071	8.000491151	2.092613243	0.000664449	ANOS1
ENSG00000128342	27.40070242	36.48648595	29.20350194	5.275275311	8.677971106	8.000491151	2.091248957	0.000545343	LIF
ENSG00000137872	349.1052457	333.5907287	324.4833549	81.76676732	74.24486391	82.0050343	2.08103112	1.85E-35	SEMA6D
ENSG00000131386	377.5207889	492.0463248	343.9523562	96.71338071	110.8851864	80.00491151	2.076030059	9.14E-31	GALNT15
ENSG00000124875	581.5037959	467.0270202	528.9078685	131.0026702	131.1337856	119.0073059	2.048234854	2.67E-47	CXCL6
ENSG00000146411	157.3003287	141.7760597	160.0784551	36.04771463	38.56876047	37.00227158	2.040950837	2.63E-16	SLC2A12
ENSG00000158050	19.28197578	19.80694952	22.71383485	6.154487863	1.928438024	7.000429757	2.032673456	0.008990135	DUSP2
ENSG00000204381	128.8847855	112.5868709	110.3243407	35.16850207	26.99813233	24.00147345	2.023770656	5.14E-12	LAYN
ENSG00000113361	92.35051557	77.14285601	86.52889465	20.22188869	22.17703727	21.00128927	2.014792234	2.78E-09	CDH6
ENSG00000253522	154.2558062	136.5637046	173.0577893	38.68535228	44.35407454	32.00196461	2.00959507	3.81E-15	MIR3142HG
ENSG00000214900	25.37102076	22.9343626	22.71383485	9.671338071	2.892657035	5.00030697	2.000900782	0.005555529	LINC01588
ENSG00000182308	24.35617993	27.10424671	16.22416775	6.154487863	7.713752095	3.000184182	1.999776451	0.006775566	DCAF4L1
ENSG00000035664	52.77172318	64.63320368	42.18283614	15.82582593	7.713752095	17.0010437	1.976214409	2.16E-05	DAPK2
ENSG00000228594	223.2649827	241.8532783	197.9348465	61.54487863	60.74579774	46.00282412	1.975090527	1.91E-20	FNDC10
ENSG00000176697	909.2973841	845.444003	868.53378	242.6626643	223.6988107	203.012463	1.968163353	4.17E-76	BDNF
ENSG00000165092	6501.07036	6719.768241	6350.139256	1684.571249	1672.919986	1661.101975	1.963268223	0	ALDH1A1
ENSG00000115009	63.93497232	43.78378314	46.50928087	13.18818828	16.3917232	10.00061394	1.961176635	2.00E-05	CCL20
ENSG00000130755	93.3653564	88.61003731	71.38633808	7.033700415	31.81922739	27.00165764	1.95650077	3.32E-06	GMFG
ENSG00000164056	213.1165744	187.6447849	153.588788	42.20220249	44.35407454	58.00356085	1.944013225	5.62E-16	SPRY1
ENSG00000092969	1081.820325	1018.494194	966.9603977	236.5081765	256.4822571	306.0187865	1.943877718	1.16E-74	TGFB2
ENSG00000021300	52.77172318	51.08108033	36.77478023	14.06740083	16.3917232	6.000368364	1.941449792	0.000105105	PLEKHB1
ENSG00000170214	36.5342699	43.78378314	27.04027958	9.671338071	11.57062814	7.000429757	1.924753067	0.000565066	ADRA1B
ENSG00000065320	22.32649827	37.52895698	41.10122496	7.033700415	8.677971106	11.00067533	1.922758332	0.001035138	NTN1
ENSG00000110328	21.31165744	20.84942054	29.20350194	7.033700415	6.749533083	5.00030697	1.920132789	0.005483917	GALNT18
ENSG00000243742	62.92013149	72.9729719	85.44728347	20.22188869	10.60640913	28.00171903	1.913428798	1.55E-06	RPLP0P2
ENSG00000082497	51.75688235	46.91119622	67.05989335	10.55055062	23.14125628	11.00067533	1.893448136	3.87E-05	SERTAD4
ENSG00000118473	587.5928408	519.1505715	558.1113705	156.4998342	133.0622236	160.009823	1.888558255	2.45E-46	SGIP1
ENSG00000257591	38.56395156	23.97683362	33.52994668	1.758425104	12.53484715	12.00073673	1.886376605	0.004582506	ZNF625
ENSG00000140873	533.8062768	454.5173678	465.0928087	125.7273949	134.9906617	133.0081654	1.885514837	3.96E-41	ADAMTS18
ENSG00000072041	173.537782	161.5830092	184.9555123	39.56456483	53.03204565	49.0030083	1.880325122	8.27E-16	SLC6A15
ENSG00000087085	47.69751903	29.18918876	52.99894797	6.154487863	12.53484715	17.0010437	1.875388899	0.000454387	ACHE
ENSG00000184368	76.11306229	70.88802985	61.65183744	26.37637656	20.24859925	10.00061394	1.871145481	3.69E-06	MAP7D2
ENSG00000179256	27.40070242	17.72200746	20.55061248	6.154487863	4.821095059	7.000429757	1.871029054	0.009782465	SMCO3
ENSG00000137474	163.3893737	219.9613867	190.3635682	50.11511546	63.63845478	43.00263994	1.870925648	1.54E-15	MYO7A
ENSG00000128645	134.9738305	135.5212335	168.7313446	27.25558911	50.13938861	45.00276273	1.849524209	1.60E-11	HOXD1
ENSG00000135914	105.5434464	99.03474758	101.6714512	25.497164	38.56876047	21.00128927	1.848084096	5.31E-09	HTR2B
ENSG00000108679	35.51942907	20.84942054	18.38739011	5.275275311	9.642190118	6.000368364	1.843273104	0.008207129	LGALS3BP
ENSG00000270607	426.2331488	394.0540483	514.8469232	108.1431439	134.9906617	130.0079812	1.841199926	1.11E-31	AC009549.1
ENSG00000147408	300.3928858	325.2509605	296.3614642	81.76676732	100.2787772	76.00466594	1.837253804	1.05E-25	CSGALNACT1
ENSG00000100767	903.2083391	746.4092554	867.4521688	246.1795145	217.9134967	241.0147959	1.835248813	2.73E-59	PAPLN
ENSG00000155966	58.86076817	44.82625417	43.26444732	16.70503849	14.46328518	10.00061394	1.830065128	7.81E-05	AFF2

ENSG00000112149	104.5286055	114.671813	89.7737282	22.85952635	34.71188443	30.00184182	1.824003839	3.98E-09	CD83
ENSG00000270547	452.6190104	382.586867	370.9926358	121.3313322	87.74393008	132.008104	1.822704107	2.88E-27	LINC01235
ENSG00000162595	483.0642353	437.8378314	405.6041937	123.0897573	115.7062814	138.0084724	1.817388359	5.92E-35	DIRAS3
ENSG00000074527	7517.940872	7601.69873	7014.248522	2003.725406	2195.52669	2100.128927	1.813574691	0	NTN4
ENSG00000188112	84.23178893	55.25096444	71.38633808	24.61795145	18.32016122	17.0010437	1.809506266	2.62E-06	C6orf132
ENSG00000171004	29.43038408	35.44401492	23.79544603	11.42976317	7.713752095	6.000368364	1.809247817	0.00327557	HS6ST2
ENSG00000144218	122.7957405	151.1582989	123.3036749	35.16850207	29.89078937	49.0030083	1.803932424	8.35E-11	AFF3
ENSG00000175264	200.9384844	193.899611	246.6073497	73.85385436	49.1751696	61.00374503	1.796977891	3.64E-16	CHST1
ENSG00000254585	22.32649827	43.78378314	31.36672431	12.30897573	4.821095059	11.00067533	1.787704786	0.003580026	MAGEL2
ENSG00000170891	276.0367059	272.0849381	261.7499063	87.04204264	55.92470269	92.00564824	1.784618398	1.71E-19	CYTL1
ENSG00000198046	37.54911073	26.06177568	31.36672431	6.154487863	8.677971106	13.00079812	1.781329941	0.002671326	ZNF667
ENSG00000149633	48.71235986	22.9343626	28.12189076	7.033700415	11.57062814	11.00067533	1.760710596	0.003314827	KIAA1755
ENSG00000143369	297.3483633	283.5521194	317.9936878	91.43810539	86.77971106	87.00534127	1.759857269	5.24E-25	ECM1
ENSG00000125740	23.3413391	31.27413081	44.34605851	9.671338071	11.57062814	8.000491151	1.755491765	0.002642018	FOSB
ENSG00000013016	144.1073979	120.9266391	144.9358985	34.28928952	40.4971985	47.00288551	1.755377543	2.25E-11	EHD3
ENSG00000100311	2192.056194	2078.687228	2179.446534	664.6846892	642.1698619	603.0370205	1.754731717	7.02E-145	PDGFB
ENSG00000135063	405.9363322	332.5482577	349.3604121	120.4521196	82.92283502	119.0073059	1.753696488	5.23E-24	FAM189A2
ENSG00000081842	35.51942907	17.72200746	25.95866839	9.671338071	6.749533083	7.000429757	1.75314371	0.008182844	PCDHA6
ENSG00000129910	23.3413391	25.01930465	33.52994668	11.42976317	6.749533083	6.000368364	1.748227379	0.00654233	CDH15
ENSG00000267534	19.28197578	43.78378314	38.93800259	7.033700415	16.3917232	7.000429757	1.746991977	0.004932987	S1PR2
ENSG00000174175	55.81624568	72.9729719	59.4886507	24.61795145	12.53484715	19.00116648	1.739381753	2.09E-05	SELP
ENSG00000188372	25.37102076	26.06177568	23.79544603	6.154487863	10.60640913	6.000368364	1.727103486	0.009027873	ZP3
ENSG00000168386	1648.101509	1618.957505	1443.950929	465.1034399	510.0718573	453.0278114	1.722213142	8.36E-94	FILIP1L
ENSG00000182107	32.47490658	34.4015439	25.95866839	13.18818828	4.821095059	10.00061394	1.722039754	0.004560227	TMEM30B
ENSG00000147676	51.75688235	56.29343547	60.57022625	12.30897573	18.32016122	21.00128927	1.71442744	4.65E-05	MAL2
ENSG00000128872	308.5116125	236.6409232	249.8521833	74.73306691	77.13752095	91.00558685	1.713617789	6.02E-19	TMOD2
ENSG00000163131	230.3688685	177.2200746	228.2199596	61.54487863	70.38798786	62.00380642	1.713547887	6.71E-16	CTSS
ENSG00000159784	106.5582872	90.69497936	89.7737282	31.65165187	31.81922739	24.00147345	1.711728508	4.31E-08	FAM131B
ENSG00000176907	780.4125986	709.9227695	758.2094394	222.4407756	242.018972	223.0136908	1.710033641	1.63E-55	TCIM
ENSG00000172738	234.4282318	250.1930465	264.9947399	76.49149201	77.13752095	76.00466594	1.706359656	3.40E-20	TMEM217
ENSG00000196562	3609.788834	3616.331993	3342.178556	1107.807815	1136.814215	1011.062069	1.698223517	4.66E-174	SULF2
ENSG00000277117	113.662173	165.7528933	102.7530624	59.78645353	27.96235134	30.00184182	1.690100366	3.02E-07	FP565260.3
ENSG00000164099	66.97949481	77.14285601	87.61050583	32.53086442	19.28438024	20.00122788	1.681342267	3.89E-06	PRSS12
ENSG00000123095	26.38586159	41.69884109	35.69316904	7.033700415	6.749533083	19.00116648	1.673212392	0.005351634	BHLHE41
ENSG00000078401	12725.08917	12474.20831	12927.41686	3869.414441	4189.531606	3936.241646	1.668551767	0	EDN1
ENSG00000158406	55.81624568	41.69884109	28.12189076	12.30897573	17.35594221	10.00061394	1.664774553	0.001399332	HIST1H4H
ENSG00000273264	28.41554325	37.52895698	27.04027958	7.912912967	10.60640913	11.00067533	1.660461517	0.004333402	AL360219.1
ENSG00000165072	239.502436	221.0038578	208.7509583	65.06172884	76.17330193	71.00435897	1.658931105	4.32E-17	MAMDC2
ENSG00000184922	58.86076817	56.29343547	62.73344862	23.7387389	12.53484715	20.00122788	1.65555865	5.63E-05	FMNL1
ENSG00000064692	183.6861903	183.4749008	182.7922899	62.42409118	55.92470269	56.00343806	1.655530482	1.30E-14	SNCAIP
ENSG00000165449	66.97949481	93.82239244	57.3253927	18.46346359	24.1054753	27.00165764	1.654084729	1.33E-05	SLC16A9
ENSG00000148200	32.47490658	32.31660184	24.87705721	7.033700415	8.677971106	13.00079812	1.651910388	0.005894215	NR6A1
ENSG00000268355	42.62331488	43.78378314	35.69316904	8.792125519	21.21281826	9.000552545	1.650500819	0.001861765	AC243960.3
ENSG00000196611	27.40070242	47.95366725	32.44833549	7.033700415	10.60640913	17.0010437	1.648069243	0.003961425	MMP1
ENSG00000272720	61.90529066	60.46331957	69.22311572	18.46346359	26.03391332	17.0010437	1.639611367	2.36E-05	AL022322.1
ENSG00000228495	1608.522716	1632.509628	1601.866162	608.4150859	464.7535637	479.0294077	1.638981178	1.13E-72	LINC01013
ENSG00000154654	66.97949481	66.71814574	76.794394	29.89322676	18.32016122	19.00116648	1.638929567	1.23E-05	NCAM2
ENSG00000163545	237.4727543	272.0849381	290.9534083	81.76676732	84.85127304	91.00558685	1.636376972	3.21E-19	NUAK2
ENSG00000127561	90.32083391	77.14285601	82.20244992	21.9803138	32.7834464	26.00159624	1.631789084	1.16E-06	SYNGR3
ENSG00000140945	1620.700806	1651.274107	1602.947773	545.9909947	547.6763987	484.0297147	1.626413461	3.05E-100	CDH13
ENSG00000085465	45.66783737	38.571428	35.69316904	7.912912967	16.3917232	15.00092091	1.618859192	0.001650179	OVGP1
ENSG00000167971	62.92013149	51.08108033	46.50928087	20.22188869	24.1054753	8.000491151	1.611474205	0.000549567	CASKIN1
ENSG00000255836	66.97949481	56.29343547	40.01961377	19.34267614	16.3917232	18.00110509	1.60367591	0.000206633	AC131206.1
ENSG00000235505	639.3497232	622.3552032	698.7208243	207.4941622	219.8419347	223.0136908	1.59256209	1.62E-43	CASP17P
ENSG00000175874	34.50458824	40.65637006	35.69316904	14.06740083	7.713752095	15.00092091	1.590413484	0.002699883	CREG2
ENSG00000130540	37.54911073	51.08108033	63.8150598	15.82582593	26.03391332	9.000552545	1.580272419	0.001244646	SULT4A1
ENSG00000152689	535.8359585	457.6447809	432.6444732	170.5672351	149.4539468	157.0096388	1.579235445	5.17E-29	RASGRP3
ENSG00000154678	176.5823045	138.6486466	146.0175097	49.2359029	53.03204565	53.00325388	1.572460238	6.21E-11	PDE1C
ENSG00000176463	67.99433564	72.9729719	110.3243407	31.65165187	30.85500838	22.00135067	1.567022898	1.04E-05	SLCO3A1
ENSG00000058085	1207.660588	1005.984541	1173.548134	412.3506868	372.1885386	370.0227158	1.551506437	1.04E-56	LAMC2
ENSG00000187372	66.97949481	86.52509525	72.46794927	25.497164	26.03391332	26.00159624	1.543408997	8.77E-06	PCDHB13
ENSG00000189134	187.7455536	209.5366765	150.3439545	63.30330373	71.35220687	54.00331527	1.537013131	2.37E-11	NKAPL
ENSG00000130203	178.6119862	180.3474877	163.3232886	58.90724098	65.5668928	56.00343806	1.533208813	2.91E-12	APOE
ENSG00000184292	1512.112837	1433.397662	1515.337268	543.3533571	512.9645143	504.0309425	1.514455113	4.03E-86	TACSTD2
ENSG00000043591	234.4282318	209.5366765	268.2395734	84.40440498	78.10173996	87.00534127	1.512729572	4.65E-15	ADRB1
ENSG00000185133	49.72720069	45.86872519	57.3253927	17.58425104	23.14125628	13.00079812	1.506830171	0.000622222	INPP5J
ENSG00000284237	36.5342699	34.4015439	32.44833549	16.70503849	14.46328518	5.00030697	1.504826486	0.009255942	AL356275.1
ENSG00000242615	43.63815571	27.10424671	33.52994668	13.18818828	9.642190118	14.00085951	1.50188218	0.006158791	AC022415.1
ENSG00000142871	23765.54257	23713.08845	25279.41657	8289.215939	8731.003152	8700.534127	1.500318984	0	CYR61
ENSG00000257800	32.47490658	29.18918876	56.24378152	13.18818828	13.49906617	15.00092091	1.499351523	0.00452422	FNBP1P1
ENSG00000147113	3911.196561	3702.857088	3722.905692	1317.060403	1293.981914	1406.08632	1.497398579	7.66E-168	CXorf36
ENSG00000204389	6953.68937	7974.903358	8000.677921	2391.458141	2830.947019	2912.178779	1.495285924	1.43E-42	HSPA1A
ENSG00000250474	42.62331488	45.86872519	30.28511313	6.154487863	16.3917232	20.00122788	1.494117176	0.006839327	WBP1LP2
ENSG00000186642	1300.011104	1238.45558	1230.873526	430.8141504	490.787477	419.0257241	1.491722489	1.44E-66	PDE2A
ENSG00000196169	137.0035121	169.9227774	154.6703992	59.78645353	49.1751696	55.00337667	1.491232206	3.78E-10	KIF19
ENSG00000111981	166.4338962	203.2818503	164.4048998	49.2359029	69.42376885	72.00442036	1.490948884	1.31E-10	ULBP1
ENSG00000109743	405.9363322	393.0115772	442.3789739	153.8621966	138.8475377	150.0092091	1.486441319	8.81E-26	BST1
ENSG00000204403	41.60847405	40.65637006	54.08055916	11.42976317	10.60640913	27.00165764	1.483863287	0.003546429	CASP12
ENSG00000116774	27.40070242	43.78378314	33.52994668	14.06740083	13.49906617	10.00061394	1.474677893	0.006876865	OLFML3
ENSG00000125378	3753.896232	3781.042415	3786.720752	1308.268277	1372.083654	1399.08589	1.473351287	7.51E-181	BMP4
ENSG00000068976	52.77172318	39.61389903	52.99894797	20.22188869	18.32016122	14.00085951	1.463820071	0.001083951	PYGM
ENSG00000182013	241.5321177	223.0887998	192.5267906	73.85385436	75.20908292	91.00558685	1.455802074	4.98E-13	PNMA8A
ENSG00000213626	229.3540277	247.0656334	211.9957919	70.33700415	89.6723681	92.00564824	1.453586107	1.10E-13	LBH
ENSG00000165152	74.08338062	115.714284	103.8346736	44.83984015	25.06969431	37.00227158	1.452971233	1.19E-05	TMEM246
ENSG00000120324	48.71235986	36.48648595	33.52994668	14.94661338	14.46328518	14.00085951	1.451992191	0.003604284	PCDHB10
ENSG00000100228	181.6565087	129.2664074	137.3646203	53.63196566	58.81735972	52.00319248	1.447632586	6.55E-09	RAB36
ENSG00000179909	288.2147959	183.4749008	240.1176826	106.3847188	74.24486391	80.00491151	1.446328173	6.48E-11	ZNF154
ENSG00000207870	52.77172318	46.91119622	33.52994668	16.70503849	19.28438024	13.00079812	1.442770891	0.002504249	MIR221
ENSG00000243710	51.75688235	62.54826163	73.54956045	35.16850207	14.46328518	19.00116648	1.442177331	0.000764728	CFAP57
ENSG00000088899	276.0367059	264.7876409	319.075299	111.6599941	88.70814909	116.0071217	1.441720972	5.92E-16	LZTS3
ENSG00000160145	511.4797786	465.9845491	426.1548061	162.6543221	180.3089552	175.0107439	1.439848323	4.38E-26	KALRN
ENSG00000178343	224.2798235	190.772198	219.5670702	66.82015394	86.77971106	81.00497291	1.438498256	1.40E-12	SHISA3
ENSG00000197822	282.1257509	265.8301119	224.9751261	93.1965305	96.42190118	96.00589382	1.437400068	3.13E-15	OCLN

ENSG00000157240	321.7045433	364.8648595	329.8914108	118.6936945	128.2411286	129.0079198	1.436105209	2.26E-20	FZD1
ENSG00000184838	54.80140485	61.5057906	51.91733679	25.497164	10.60640913	26.00159624	1.435670582	0.001100015	PRR16
ENSG00000175746	544.969526	501.4285641	512.6837008	147.7077087	232.3767818	199.0122174	1.432223272	6.04E-22	C15orf54
ENSG00000117643	40.59363322	46.91119622	37.85639141	12.30897573	15.42750419	19.00116648	1.429317294	0.0030648	MAN1C1
ENSG00000137266	489.1532803	462.857136	555.9481481	153.8621966	213.0924016	194.0119104	1.429012555	3.15E-23	SLC22A23
ENSG00000164932	1698.84355	1682.548238	1823.596455	662.9262641	675.9175273	598.0367136	1.425126007	2.41E-80	CTHRC1
ENSG00000166257	198.9088028	194.9420821	168.7313446	58.02802842	71.35220687	81.00497291	1.423639024	6.56E-11	SCN3B
ENSG00000233822	66.97949481	70.88802985	57.3253927	25.497164	17.35594221	30.00184182	1.423142372	0.000212724	HIST1H2BN
ENSG00000108387	63.93497232	50.0386093	36.77478023	21.9803138	24.1054753	10.00061394	1.422228466	0.003098747	SEPTIN4
ENSG00000183049	90.32083391	94.86486347	59.48861507	43.08141504	26.03391332	22.00135067	1.418159588	0.000146323	CAMK1D
ENSG00000156345	46.6826782	50.0386093	36.77478023	16.70503849	18.32016122	15.00092091	1.416129657	0.002238864	CDK20
ENSG00000168140	39.57879239	53.16602238	34.61155786	21.10110124	12.53484715	14.00085951	1.412640085	0.004380007	VASN
ENSG00000136367	104.5286055	70.88802985	80.03922755	35.16850207	35.67610344	25.00153485	1.411899162	2.99E-05	ZFHX2
ENSG00000148848	52.77172318	59.42084855	31.36672431	12.30897573	18.32016122	24.00147345	1.402666321	0.003917335	ADAM12
ENSG00000138311	37.54911073	34.4015439	46.50928087	8.792125519	21.21281826	15.00092091	1.402530147	0.007588424	ZNF365
ENSG00000146374	110.6176505	75.05791395	117.895619	29.01401421	40.4971985	46.00282412	1.398786548	1.30E-05	RSPO3
ENSG00000204642	74.08338062	66.71814574	74.63117163	33.41007697	19.28438024	29.00178042	1.395275982	0.000120751	HLA-F
ENSG00000135919	166.4338962	215.7915026	164.4048998	81.76676732	64.60267379	61.00374503	1.3947315	9.93E-10	SERPINE2
ENSG00000204388	2753.263173	3099.266364	3093.407984	945.1534933	1212.023298	1252.076865	1.392320235	2.78E-24	HSPA1B
ENSG00000198342	96.4098789	71.93050087	49.75411442	24.61795145	26.03391332	33.002026	1.387371731	0.000311401	ZNF442
ENSG00000277639	61.90529066	50.0386093	57.3253927	25.497164	26.99813233	12.00073673	1.386179102	0.001311355	AC007906.2
ENSG00000169604	216.1610969	189.7297269	154.6703992	53.63196566	81.958616	80.00491151	1.383935476	4.30E-09	ANTXR1
ENSG00000100344	47.69751903	60.46331957	70.3047269	24.61795145	22.17703727	22.00135067	1.372302625	0.000547488	PNPLA3
ENSG00000163739	5228.459959	4508.687192	5355.056968	1921.079426	1912.0463	2029.124568	1.36447115	4.79E-110	CXCL1
ENSG00000047617	229.3540277	241.8532783	244.4441274	86.16283008	85.81549205	107.0065692	1.361224897	2.62E-13	ANO2
ENSG00000229689	37.54911073	35.44401492	56.24378152	24.61795145	9.642190118	16.0009823	1.353425314	0.009204518	AC009237.3
ENSG00000167103	56.83108651	32.31660184	36.77478023	14.06740083	11.57062814	24.00147345	1.34963884	0.009683084	PIP5KL1
ENSG00000060656	255.7398893	271.0424671	297.4430754	118.6936945	83.88705403	121.0074287	1.347629638	3.41E-13	PTPRU
ENSG00000184545	740.8338062	770.3860891	829.5957774	305.9659681	301.8005507	317.0194619	1.3397604	3.29E-39	DUSP8
ENSG00000169429	4844.850125	4116.718086	4712.579925	1772.492505	1794.411581	1841.113026	1.33851562	3.26E-110	CXCL8
ENSG00000152128	120.7660588	139.6911176	82.20244992	44.83984015	49.1751696	42.00257854	1.333810254	6.87E-06	TMEM163
ENSG00000269388	43.63815571	57.33590649	27.04027958	17.58425104	14.46328518	19.00116648	1.327999746	0.008692403	AC018755.3
ENSG00000254837	49.72720069	56.29343547	50.83572561	19.34267614	15.42750419	28.00171903	1.324928403	0.002092069	AP001372.2
ENSG00000189337	129.8996263	174.0926615	153.588788	54.51117822	68.45954984	60.00368364	1.323631779	4.16E-08	KAZN
ENSG00000139926	3548.898384	3416.177556	3563.908848	1371.571581	1429.936795	1412.086688	1.321580986	2.35E-142	FRMD6
ENSG00000105696	59.875609	39.61389903	33.52994668	13.18818828	18.32016122	22.00135067	1.321474451	0.007339815	TMEM59L
ENSG00000165801	289.2296367	236.6409232	218.485459	101.988656	97.38612019	100.0061394	1.314548838	2.67E-12	ARHGEF40
ENSG00000075275	532.791436	559.8069416	491.0514771	201.3396744	206.3428685	230.0141206	1.314018891	1.05E-25	CELSR1
ENSG00000169860	48.71235986	51.08108033	38.93800259	15.82582593	15.42750419	25.00153485	1.308094149	0.004690892	P2RY1
ENSG00000136274	261.8289343	271.0424671	307.177576	131.0026702	90.63658711	117.0071831	1.307954644	7.30E-13	NACAD
ENSG00000149243	142.0777163	215.7915026	131.9565643	58.90724098	75.20908292	64.00392921	1.307541436	3.99E-07	KLHL35
ENSG00000118971	130.9144671	187.6447849	140.6094538	70.33700415	53.03204565	62.00380642	1.306372438	1.80E-07	CCND2
ENSG00000136732	51.75688235	81.31274012	72.46794927	24.61795145	25.06969431	34.00208739	1.298810862	0.000597254	GYPC
ENSG00000164684	318.6600208	268.957525	261.7499063	122.2105447	91.60080612	132.008104	1.296899418	1.85E-12	ZNF704
ENSG00000171435	272.9921834	247.0656334	180.6290676	91.43810539	81.958616	114.0069989	1.288413584	1.88E-09	KSR2
ENSG00000205502	429.2776713	366.9498016	414.2570831	185.5138484	149.4539468	160.009823	1.288056657	1.74E-18	C2CD4B
ENSG00000138080	104.5286055	95.9073345	88.69211701	32.53086442	42.42563652	44.00270133	1.285757837	1.83E-05	EMILIN1
ENSG00000166016	140.0480346	88.61003731	121.1404525	62.42409118	43.38985553	39.00239436	1.267273867	2.69E-05	ABTB2
ENSG00000129946	308.5116125	386.7567511	394.7880818	142.4324334	173.5594221	137.008411	1.266459075	6.88E-15	SHC2
ENSG00000234745	3764.04464	3924.903417	3771.578195	1555.327004	1579.390741	1633.100256	1.265713053	6.69E-139	HLA-B
ENSG00000113555	693.1362872	598.3783696	617.5999856	277.8311664	243.94741	273.0167605	1.26401828	1.62E-27	PCDH12
ENSG00000184371	1938.345986	1896.254798	1955.553019	778.982321	872.6182057	761.0467208	1.263085788	1.27E-72	CSF1
ENSG00000008300	174.5526228	159.4980672	170.8945669	58.02802842	73.2806449	80.00491151	1.260704793	1.75E-08	CELSR3
ENSG00000211448	71.03885813	42.74131211	70.3047269	21.10110124	27.96235134	28.00171903	1.259583017	0.001913267	DIO2
ENSG00000158683	114.6770138	135.5212335	127.6301196	52.75275311	42.42563652	64.00392921	1.248219977	2.49E-06	PKD1L1
ENSG00000113448	1015.855671	949.6911057	998.327122	420.2635998	422.3279272	406.0249259	1.24686746	7.24E-45	PDE4D
ENSG00000111012	63.93497232	65.67567471	63.8150598	27.25558911	15.42750419	39.00239436	1.24630117	0.002230516	CYP27B1
ENSG00000074590	2209.308488	2288.223905	2105.896974	912.6226288	961.3263548	911.0559299	1.245816937	1.16E-83	NUAK1
ENSG00000057657	133.9589896	107.3745158	131.9565643	51.87354056	50.13938861	56.00343806	1.240903116	1.70E-06	PRDM1
ENSG00000076826	47.69751903	47.95366725	50.83572561	22.85956235	25.06969431	14.00085951	1.237751616	0.005356702	CAMSAP3
ENSG00000088881	77.12790312	123.0115812	89.7737282	49.2359029	41.46141751	32.00196461	1.23583332	0.000142064	EBF4
ENSG00000133800	1079.790644	1035.17373	1063.223793	415.867537	451.2544975	489.0300216	1.230432409	6.14E-43	LYVE1
ENSG00000160867	63.93497232	61.5057906	74.63117163	24.61795145	33.74766541	27.00165764	1.23013812	0.000783654	FGFR4
ENSG00000099282	507.4204152	568.1467098	564.6010376	243.5418769	247.804286	208.0127699	1.228056646	4.49E-23	TSPAN15
ENSG00000133805	73.06853979	95.9073345	72.46794927	30.77243932	34.71188443	38.00233297	1.224926664	0.000235938	AMPD3
ENSG00000122641	1727.259093	1577.258664	1589.968439	716.5582298	693.2734695	686.0421162	1.223353623	3.73E-63	INHBA
ENSG00000164442	4605.347689	4635.868658	4829.393933	1971.194541	2133.816673	1926.118245	1.222109671	9.73E-128	CITED2
ENSG00000004139	194.8494395	199.1119662	201.1796801	95.83416815	89.6723681	69.00423618	1.222014786	2.69E-09	SARM1
ENSG00000261468	154.2558062	125.0965233	126.5485084	65.94094139	44.35407454	64.00392921	1.218696326	2.82E-06	AC096921.2
ENSG00000133401	66.97949481	68.80308779	68.14150454	30.77243932	27.96235134	29.00178042	1.215716173	0.000567374	PDZD2
ENSG00000091879	2977.542997	2790.69494	2907.37086	1193.970645	1251.556277	1295.079505	1.2144987	8.38E-98	ANGPT2
ENSG00000128165	100.4692422	114.671813	86.52889465	35.16850207	53.03204565	43.00263994	1.204774426	6.70E-05	ADM2
ENSG00000282057	65.96465398	56.29343547	56.24378152	23.7387389	30.85500838	23.00141206	1.20313019	0.001960413	AC092807.3
ENSG00000117586	738.8041246	688.0308779	723.5978815	283.1064417	300.8363317	353.0216721	1.200743788	2.36E-27	TNFSF4
ENSG00000262576	153.2409654	156.3706541	117.895619	59.78645353	62.67423577	64.00392921	1.198788431	9.47E-07	PCDHGA4
ENSG00000188042	247.6211626	274.1698801	288.7901859	114.2976317	127.2769096	113.0069375	1.192569756	3.11E-12	ARL4C
ENSG00000064547	99.45440139	59.42084855	67.05989335	36.92692718	19.28438024	43.00263994	1.188434688	0.002796487	LPAR2
ENSG00000211445	163.3893737	129.2664074	120.0588413	59.78645353	62.67423577	59.00362224	1.186532612	2.10E-06	GPX3
ENSG00000247746	62.92013149	72.9729719	64.89667099	35.16850207	31.81922739	21.00128927	1.185029837	0.001475096	USP51
ENSG00000215105	71.03885813	67.76061676	75.71278282	32.53086442	32.7834464	29.00178042	1.184029034	0.000572212	TTC3P1
ENSG00000144810	8878.842426	8418.996015	8559.870903	3790.285311	3762.382584	3841.235814	1.182458248	2.93E-204	COL8A1
ENSG00000182492	15483.42655	15519.26618	15032.23222	6828.84389	6920.199848	6605.405507	1.17730333	1.77E-261	BGN
ENSG00000102032	158.3151696	124.0540522	143.8542874	73.85385436	65.5668928	49.0030083	1.17379284	3.59E-06	RENBP
ENSG00000172031	141.0628754	139.6911176	164.4048998	79.12912967	63.63845478	54.00331527	1.172840624	1.49E-06	EPHX4
ENSG00000158373	93.3653564	72.9729719	74.63117163	31.65165187	34.71188443	41.00251715	1.170042354	0.000413749	HIST1H2BD
ENSG00000133256	62.92013149	56.29343547	63.8150598	26.37637656	26.03391332	29.00178042	1.16963882	0.001870134	PDE6B
ENSG00000156466	367.3723806	332.5482577	343.9523562	149.4661338	161.024575	154.0094547	1.168966615	2.01E-15	GDF6
ENSG00000143603	108.5879689	90.69497936	78.95761637	37.80613973	53.03204565	33.002026	1.168927881	0.000277673	KCNN3
ENSG00000171223	1440.059138	1492.818511	1542.377547	694.577916	684.5954984	610.0374503	1.168470278	1.35E-51	JUNB
ENSG00000156804	174.5526228	204.3243213	164.4048998	86.16283008	81.958616	74.00454315	1.164546202	4.54E-08	FBXO32
ENSG00000151014	676.8988339	722.4324218	750.6381611	332.3423446	331.6913401	295.0181112	1.163211627	6.18E-28	NOCT
ENSG00000158528	43.63815571	62.54826163	49.75411442	23.7387389	29.89078937	16.0009823	1.160558763	0.008623524	PPP1R9A
ENSG00000144152	126.8551038	126.1389943	107.0795071	42.20220249	57.85314071	62.00380642	1.156847399	1.99E-05	FBLN7

ENSG00000104951	258.7844118	246.0231624	230.383182	100.2302309	99.31455822	131.0080426	1.156235099	4.82E-10	IL4I1
ENSG00000242732	508.4352561	440.9652445	494.2963107	208.3733748	214.0566206	226.013875	1.155637804	1.75E-19	RTL5
ENSG00000213949	251.680526	265.8301119	188.2003459	112.5392066	100.2787772	104.006385	1.155230969	4.85E-09	ITGA1
ENSG00000234465	60.89044983	57.33590649	103.8346736	39.56456483	24.1054753	36.00221018	1.15174544	0.00329924	PINLYP
ENSG00000197646	543.9546851	494.1312669	574.3355382	228.5952635	280.5877324	218.0133839	1.148836032	1.89E-18	PDCD1LG2
ENSG00000206538	343.0162007	272.0849381	316.9120766	135.398733	145.5970708	140.0085951	1.147264726	1.83E-12	VGLL3
ENSG00000163762	1767.852727	1567.876425	1557.520104	693.6987034	769.4467714	754.046291	1.143227029	1.76E-48	TM4SF18
ENSG00000137801	282359.1643	266306.5212	273726.5869	124533.4243	122799.0765	125189.6854	1.14250335	0	THBS1
ENSG00000118523	56612.89573	55921.27331	56927.35979	25265.05189	25948.09783	25692.57727	1.139838094	0	CTGF
ENSG00000279117	2132.180585	2173.552092	2013.960023	941.6366431	945.8988506	982.0602888	1.139555319	4.64E-71	AP001972.5
ENSG00000243243	72.05369896	56.29343547	107.0795071	53.63196566	23.14125628	30.00184182	1.132663927	0.006103011	AC073130.2
ENSG00000167992	1220.853519	1242.625464	1273.056363	563.5752458	612.2790725	530.0325388	1.130782032	2.86E-43	VWCE
ENSG00000128590	1483.697294	1641.891868	1519.663712	693.6987034	729.9137919	699.0429144	1.130055606	3.11E-52	DNAJB9
ENSG00000175155	1730.303616	1559.536657	1527.234991	763.156495	686.5239364	753.0462296	1.128817765	1.27E-47	YPEL2
ENSG00000105290	307.4967716	274.1698801	307.177576	133.6403079	126.3126905	147.0090249	1.12779326	3.35E-12	APLP1
ENSG00000132561	240.5172768	258.5328147	234.7096267	91.43810539	138.8475377	107.0065692	1.123821982	6.50E-09	MATN2
ENSG00000099860	816.9468685	782.8957414	841.4935005	350.8058082	423.2921462	348.0213651	1.121809523	6.28E-27	GADD45B
ENSG00000111339	1119.369436	1077.915042	1062.142182	519.6146182	500.4296671	478.0293463	1.120781717	1.98E-40	ART4
ENSG00000100031	50.74204152	68.80308779	75.71278282	29.01401421	35.67610344	25.00153485	1.120452632	0.00340617	GGT1
ENSG00000164692	181.6565087	173.0501905	189.281957	95.83416815	84.85127304	69.00423618	1.119734795	1.65E-07	COL1A2
ENSG00000138028	52.77172318	65.67567471	58.40700389	36.92692718	26.03391332	18.00110509	1.119238449	0.007066101	CGREF1
ENSG00000115266	112.6473322	77.14285601	137.3646203	46.59826525	53.03204565	51.00313109	1.11922023	0.000235299	APC2
ENSG00000090339	3516.423478	3433.899563	3681.804467	1594.891569	1625.673254	1681.103203	1.11738864	4.23E-100	ICAM1
ENSG00000100292	28657.07537	30341.11924	30932.99823	13233.02812	14042.88569	14243.87443	1.115143898	7.50E-191	HMOX1
ENSG00000237238	56.83108651	57.33590649	55.16217034	31.65165187	14.46328518	32.00196461	1.114359254	0.009668982	BMS1P10
ENSG00000101335	1074.716439	1063.320448	1070.795071	531.0443813	522.6067044	430.0263994	1.111219702	8.94E-33	MYL9
ENSG00000138449	2429.528948	2474.826218	2443.359663	1043.625299	1260.234248	1099.067472	1.111211802	1.41E-58	SLC40A1
ENSG00000090530	198.9088028	205.3667923	226.0567373	96.71338071	107.9925293	87.00534127	1.110491551	1.12E-08	P3H2
ENSG00000177606	8750.972481	8618.107981	9180.715722	4102.405767	4183.746292	4112.252452	1.09849999	4.92E-172	JUN
ENSG00000130475	60.89044983	78.18532704	57.3253927	28.13480166	34.71188443	29.00178042	1.097784724	0.003142953	FCHO1
ENSG00000187566	135.9886713	149.0733569	150.3439545	49.2359029	81.958616	73.00448176	1.096607275	1.49E-05	NHLRC1
ENSG00000223547	423.1886263	341.9304969	383.97197	152.1037715	206.3428685	180.0110509	1.095905111	2.67E-12	ZNF844
ENSG00000162654	447.5448062	447.2200706	471.5824758	200.4604618	227.5556868	212.0130155	1.094860925	1.27E-17	GBP4
ENSG00000179044	127.8699446	167.8378354	162.2416775	72.09542925	76.17330193	66.004052	1.094376712	3.20E-06	EXOC3L1
ENSG00000160323	72.05369896	94.86486347	86.52889465	28.13480166	42.42563652	49.0030083	1.089286862	0.001287223	ADAMTS13
ENSG00000183117	207.0275294	200.1544372	180.6290676	82.64597988	80.99439699	114.0069989	1.085560062	2.88E-07	CSMD1
ENSG00000234964	212.1017336	168.8803064	176.3026228	85.28361753	93.52924415	84.00515709	1.085135579	2.22E-07	FABP5P7
ENSG00000105929	79.15758478	60.46331957	91.93695056	37.80613973	48.21095059	23.00141206	1.083576545	0.004002499	ATP6V0A4
ENSG00000167889	262.8437751	248.1081045	276.8924629	108.1431439	122.4558145	142.0087179	1.082958893	7.81E-10	MGAT5B
ENSG00000188659	56.83108651	50.0386093	68.14150454	35.16850207	23.14125628	24.00147345	1.082209641	0.007690788	SAXO2
ENSG00000119630	2242.798235	2071.389931	2199.997146	1004.939947	1011.465743	1069.06563	1.078641899	3.85E-64	PGF
ENSG00000090661	97.42471973	114.671813	109.2427295	62.42409118	48.21095059	41.00251715	1.078232719	0.000178504	CERS4
ENSG00000198108	88.29115225	66.71814574	111.4059519	37.80613973	40.4971985	48.00294691	1.078141701	0.00111691	CHSY3
ENSG00000198855	459.7228962	438.8803024	431.5628621	188.1514861	208.2713066	235.0144276	1.077444718	1.02E-15	FICD
ENSG00000175274	2003.295799	2078.687228	2037.755469	984.7180581	1007.608867	908.0557457	1.076605461	2.11E-61	TP53I11
ENSG00000168672	487.1235986	518.1081005	520.2549791	252.3340024	259.3749142	211.0129541	1.076090316	1.45E-17	FAM84B
ENSG00000026103	402.8918097	364.8648595	322.3201326	173.2048727	194.7722404	149.0091477	1.076065727	8.53E-12	FAS
ENSG00000166922	67.99433564	69.84555882	54.08055916	23.7387389	36.64032245	31.00190321	1.07505819	0.004833884	SCG5
ENSG00000164659	2026.637138	1975.482596	1957.716241	988.2349083	948.7915076	899.0551931	1.070573633	5.57E-61	KIAA1324L
ENSG00000179598	96.4098789	91.73745039	100.58984	38.68535228	48.21095059	51.00313109	1.069344051	0.00027407	PLD6
ENSG00000179546	155.2706471	151.1582989	176.3026228	87.92125519	76.17330193	66.004052	1.065253778	2.62E-06	HTR1D
ENSG00000105464	327.7935882	265.8301119	287.7085747	135.398733	161.024575	125.0076742	1.064651768	5.38E-10	GRIN2D
ENSG00000010626	58.86076817	93.82239244	72.46794927	36.04771463	38.56876047	33.002026	1.063901825	0.002622088	LRRC23
ENSG00000130513	6488.89227	6895.945844	6917.985127	3071.968656	3333.305124	3315.203521	1.062978308	8.98E-117	GDF15
ENSG00000230257	118.7363772	97.99227655	113.5691742	62.42409118	43.38985553	52.00319248	1.062582096	0.000155882	NFE4
ENSG00000165246	911.3270658	817.2972853	743.0668828	405.3169864	390.5086998	388.0238208	1.062145748	1.49E-24	NLGN4Y
ENSG00000155465	327.7935882	344.015439	341.7891339	133.6403079	167.7741081	186.0114193	1.059525556	3.15E-11	SLC7A7
ENSG00000124212	150.1964429	199.1119662	160.0784551	78.24991712	77.13752095	90.00552545	1.054856925	2.48E-06	PTGIS
ENSG00000067445	128.8847855	103.2046317	112.487563	45.7190527	61.71001676	59.00362224	1.053537905	0.000118195	TRO
ENSG00000121653	311.556135	297.1042427	300.6879089	145.9492836	152.3466039	143.0087793	1.043125852	1.43E-11	MAPK8IP1
ENSG00000050165	5804.88955	5557.413046	5743.355382	2761.606625	2814.555295	2725.167298	1.043028529	6.50E-132	DKK3
ENSG00000095015	801.7242561	767.258676	721.4346591	362.2355714	334.5839971	418.0256627	1.040082545	5.87E-22	MAP3K1
ENSG00000241717	394.7730831	403.4362875	400.1961377	175.8425104	184.1658313	224.0137522	1.039344657	2.62E-13	VWFP1
ENSG00000159261	90.32083391	117.7992261	98.42661766	41.32298994	37.60454146	71.00435897	1.036253189	0.001350748	CLDN14
ENSG00000174080	920.4606332	1079.999984	1063.223793	416.7467496	559.2470269	520.0319248	1.035646118	3.12E-21	CTSF
ENSG00000232531	62.92013149	67.76061676	56.24378152	32.53086442	23.14125628	36.00221018	1.028426233	0.007399481	AC027612.3
ENSG00000157570	3079.02708	3201.428524	2980.920421	1497.298976	1589.99715	1457.089451	1.027189765	9.20E-74	TSPAN18
ENSG00000105810	1654.190554	1401.08106	1300.096642	748.2098816	671.0964322	723.0443878	1.023675086	8.14E-29	CDK6
ENSG00000180573	207.0275294	208.4942054	164.4048998	94.9549556	107.9925293	83.0050957	1.020154479	1.35E-06	HIST1H2AC
ENSG00000063180	174.5526228	158.4555961	154.6703992	85.28361753	78.10173996	77.00472733	1.019674072	2.70E-06	CA11
ENSG00000167191	445.5151246	415.9459398	429.3996397	218.0447129	187.0584883	233.0143048	1.016749558	6.15E-14	GPRC5B
ENSG00000099953	220.2204602	244.9806914	209.8325695	94.07574305	123.4200335	117.0071831	1.015816604	1.18E-07	MMP11
ENSG00000123700	689.0769239	702.6254723	700.8840467	342.8928952	343.2619682	349.0214265	1.01552954	4.73E-24	KCNJ2
ENSG00000237172	328.8084291	341.9304969	348.278801	142.4324334	164.881451	198.012156	1.014920975	1.64E-10	B3GNT9
ENSG00000163874	476.9751903	444.0926576	495.3779219	227.7160509	289.2657035	184.0112965	1.014005401	3.38E-11	ZC3H12A
ENSG00000254486	152.2261246	125.0965233	161.1600663	80.00834222	48.21095059	89.00546406	1.012938161	0.000167302	LINC02547
ENSG00000134070	676.8988339	614.015435	620.8448191	285.7440794	321.0849309	342.0209967	1.012764772	3.31E-19	IRAK2
ENSG00000091656	95.39503806	77.14285601	54.08055916	35.16850207	45.31829356	32.00196461	1.01228821	0.006038279	ZFHX4
ENSG00000196632	104.5286055	88.61003731	112.487563	58.90724098	48.21095059	44.00270133	1.012222203	0.000505041	WNK3
ENSG00000118503	900.1638166	853.7837712	922.6143392	441.364701	483.0737249	404.0248031	1.010079611	1.54E-25	TNFAIP3
ENSG00000120129	2789.797443	2744.826214	2714.84407	1310.026702	1403.902881	1385.085031	1.009710854	3.77E-73	DUSP1
ENSG00000159167	75.09822146	67.76061676	133.0381755	44.83984015	49.1751696	43.00263994	1.0077856	0.004179495	STC1
ENSG00000073756	510.4649377	487.8764407	546.2136475	296.29463	246.840067	224.0137522	1.006615047	2.28E-14	PTGS2
ENSG00000109472	310.5412941	380.5019249	316.9120766	162.6543221	189.9511453	149.0091477	1.006555774	4.61E-10	CPE
ENSG00000276180	218.1907786	178.2625456	165.486511	103.7470811	86.77971106	89.00546406	1.006362679	2.40E-06	HIST1H4I
ENSG00000204103	530.7617543	573.3590649	576.4987606	257.6092777	341.3335302	238.0146118	1.005764261	1.75E-13	MAFB
ENSG00000165194	116.7066955	92.77992141	80.03922755	53.63196566	33.74766541	57.00349945	1.004403348	0.001930727	PCDH19
ENSG00000253953	375.4911073	351.3127361	306.0959648	203.0980995	163.917232	147.0090249	1.004211567	1.10E-09	PCDHGB4
ENSG00000163513	17991.09824	17370.69473	17674.60834	8661.122849	9085.835748	8712.534864	1.00328897	1.20E-197	TGFBR2
ENSG00000145632	6986.164277	6984.555882	7164.592477	3515.091782	3584.966286	3449.211748	1.002377768	6.90E-143	PLK2
ENSG00000158042	1022.959557	999.729715	963.7155641	1921.958638	2036.430553	2019.123954	-1.000500653	1.18E-54	MRPL17
ENSG00000100749	311.556135	320.0386053	279.0556852	619.8448491	630.5992337	574.0352401	-1.002249083	8.65E-19	VRK1
ENSG00000172244	64.94981315	44.82625417	56.24378152	99.35101836	121.4915955	113.0069375	-1.006047799	0.000340611	C5orf34

ENSG00000183763	69.00917647	92.77992141	67.05989335	163.5335346	159.0961369	137.008411	-1.00676159	2.44E-05	TRAIP
ENSG00000134955	42.62331488	44.82625417	47.59089206	81.76676732	87.74393008	102.0062622	-1.006835546	0.000970838	SLC37A2
ENSG00000149548	118.7363772	120.9266391	133.0381755	221.5615631	260.3391332	268.0164536	-1.00806391	1.75E-08	CCDC15
ENSG00000205403	70.0240173	66.71814574	83.2840611	144.1908585	153.3108229	145.0089021	-1.009066755	1.39E-05	CFI
ENSG00000111684	147.1519204	123.0115812	122.2220637	269.0390409	263.2317902	258.0158396	-1.009319056	3.58E-09	LPCAT3
ENSG00000134363	45.66783737	64.63320368	85.44728347	130.1234577	130.1695666	133.0081654	-1.009712293	0.000219131	FST
ENSG00000123473	380.5653114	418.0308819	398.0329154	764.9149201	824.4072551	834.0512025	-1.017650599	2.45E-25	STIL
ENSG00000074370	27.40070242	44.82625417	36.77478023	75.61227946	80.03017798	65.00399061	-1.019376602	0.004232934	ATP2A3
ENSG00000119969	318.6600208	282.5096484	259.5866839	599.6229604	580.4598451	568.0348718	-1.021352482	3.37E-18	HELLS
ENSG00000186767	113.662173	105.2895737	83.2840611	201.3396744	218.8777157	194.0119104	-1.021388995	4.09E-07	SPIN4
ENSG00000136824	760.115782	665.0965153	688.9863236	1424.324334	1441.507423	1428.087671	-1.021680367	6.06E-41	SMC2
ENSG00000138658	123.8105813	112.5868709	98.42661766	247.9379396	203.4502115	229.0140592	-1.022494867	8.87E-08	ZGRF1
ENSG00000006634	326.7787474	318.9961343	270.4027958	620.7240616	625.7781387	617.0378801	-1.023305978	1.57E-19	DBF4
ENSG00000159259	240.5172768	259.5752858	250.9337945	487.9629663	549.6048367	489.0300216	-1.023359196	9.49E-17	CHAF1B
ENSG00000119640	72.05369896	82.35521114	57.3253927	130.1234577	150.4181658	151.0092705	-1.024858755	2.82E-05	ACYP1
ENSG00000021645	115.6918547	108.4169868	134.1197867	252.3340024	242.983191	233.0143048	-1.025253642	1.07E-08	NRXN3
ENSG00000188229	5013.313703	5001.775988	5092.22545	10200.62403	10583.26787	10054.61725	-1.029544799	1.07E-172	TUBB4B
ENSG00000115602	13621.19363	13126.79517	14436.26446	27355.81934	29130.02057	27594.69404	-1.029726422	4.83E-155	IL1RL1
ENSG00000117650	197.8939619	260.6177568	199.0164577	420.2635998	457.0398116	469.0287938	-1.033145112	4.03E-13	NEK2
ENSG00000138778	790.5610069	775.5984442	723.5978815	1524.554565	1541.7862	1633.100256	-1.036473627	7.88E-46	CENPE
ENSG00000197635	256.7547301	234.5559811	228.2199596	502.9095797	492.715915	481.0295305	-1.036814647	3.18E-17	DPP4
ENSG00000211772	660.6613806	697.4131172	736.5772157	1447.18386	1383.654282	1467.090065	-1.03750413	1.07E-42	TRBC2
ENSG00000152760	44.65299654	44.82625417	35.69316904	88.80046774	68.45954984	100.0061394	-1.037731203	0.001540189	TCTEX1D1
ENSG00000120334	160.3448512	146.9884148	125.4668972	279.5895915	341.3335302	270.0165764	-1.040170805	2.16E-09	CENPL
ENSG00000128951	532.791436	552.5096444	536.4791468	1114.841516	1209.130641	1011.062069	-1.040434381	1.40E-30	DUT
ENSG00000146147	40.59363322	33.35907287	42.18283614	81.76676732	79.06595897	78.00478873	-1.040974299	0.001385549	MLIP
ENSG00000156802	663.7059031	580.6563621	571.0907047	1113.962303	1357.620369	1277.0784	-1.044756198	1.70E-28	ATAD2
ENSG00000000460	94.38019723	101.1196896	105.9978959	189.9099112	199.5933354	233.0143048	-1.045371301	1.17E-07	C1orf112
ENSG00000132967	370.4169031	358.6100333	441.2973627	873.9372766	776.1963045	773.0474575	-1.051640957	2.22E-22	HMGB1P5
ENSG00000249565	77.12790312	57.33590649	68.14150454	132.7610953	137.8833187	150.0092091	-1.052525647	1.51E-05	SERBP1P5
ENSG00000125319	62.92013149	84.4401532	82.20244992	155.6206217	165.84567	155.0095161	-1.054819174	3.61E-06	C17orf53
ENSG00000146263	208.0423702	240.8108073	247.6889609	443.1231261	498.5012291	506.0310653	-1.055472245	7.19E-16	MMS22L
ENSG00000121957	253.7102076	203.2818503	262.8315175	472.1371404	543.8195227	481.0295305	-1.056363288	4.78E-15	GPSM2
ENSG00000131470	65.96465398	90.69497936	81.12083873	160.895897	173.5594221	160.009823	-1.057122978	2.38E-06	PSMC3IP
ENSG00000232759	61.90529066	59.42084855	64.89667099	126.6066075	139.8117567	121.0074287	-1.057295651	2.09E-05	AC002480.1
ENSG00000123975	310.5412941	321.0810764	348.278801	718.3166549	725.0926969	594.036468	-1.057452551	1.03E-19	CKS2
ENSG00000138376	185.715872	189.7297269	168.7313446	398.283286	370.2601005	367.0225316	-1.061274465	5.96E-14	BARD1
ENSG00000133863	93.3653564	69.84555882	68.14150454	162.6543221	136.9190997	184.0112965	-1.061566083	9.20E-06	TEX15
ENSG00000119403	785.4868028	846.486474	837.1670557	1770.734079	1744.272192	1643.10087	-1.063509156	9.03E-52	PHF19
ENSG00000139832	48.71235986	29.18918876	44.34605851	86.16283008	76.17330193	94.00577103	-1.067613022	0.001282301	RAB20
ENSG00000077152	163.3893737	199.1119662	178.4658452	396.5248609	390.5086998	347.0213037	-1.06898446	3.31E-13	UBE2T
ENSG00000205208	350.1200865	282.5096484	277.9740741	621.6032742	658.5615851	643.0394763	-1.07719539	4.14E-20	C4orf46
ENSG00000115896	365.342699	325.2509605	309.3407984	674.3560273	716.4147258	723.0443878	-1.078573654	8.48E-24	PLCL1
ENSG00000101868	280.0960692	262.7026988	270.4027958	560.0583955	642.1698619	516.0316793	-1.079250137	2.36E-18	POLA1
ENSG00000185480	173.537782	170.9652484	124.3852861	320.9125814	334.5839971	339.0208125	-1.082645234	3.38E-11	PARPBP
ENSG00000119514	31.46006574	28.14671773	19.4690013	61.54487863	48.21095059	58.00356085	-1.083087392	0.008881001	GALNT12
ENSG00000168496	813.902346	788.1080965	814.4532209	1732.048727	1706.667651	1686.10351	-1.084795117	1.61E-58	FEN1
ENSG00000173207	289.2296367	301.2741268	335.2994668	650.6172884	674.9533083	642.0394149	-1.08855814	1.79E-23	CKS1B
ENSG00000164032	1964.731848	2022.393793	2168.630422	4406.61331	4439.26433	4251.260986	-1.089764868	1.98E-102	H2AFZ
ENSG00000058804	565.2663426	623.3976742	578.661983	1254.636312	1285.303943	1243.076313	-1.09805069	1.21E-43	NDC1
ENSG00000279348	23.3413391	54.20849341	30.28511313	72.97464181	61.71001676	97.00595521	-1.102769902	0.005567779	AC012513.3
ENSG00000197594	80.17242561	79.22779806	63.8150598	151.2245589	182.2373932	147.0090249	-1.104456228	1.74E-06	ENPP1
ENSG00000223865	37.54911073	29.18918876	35.69316904	81.76676732	66.53111182	72.00442036	-1.105821745	0.001395035	HLA-DPB1
ENSG00000138346	126.8551038	116.756755	160.0784551	291.0193547	279.6235134	300.0184182	-1.110285315	9.05E-11	DNA2
ENSG00000151470	69.00917647	77.14285601	67.05989335	162.6543221	159.0961369	141.0086565	-1.118590333	9.36E-07	C4orf33
ENSG00000231607	64.94981315	47.95366725	40.01961377	122.2105447	112.8136244	98.0060166	-1.12139833	0.000113576	DLEU2
ENSG00000253669	27.40070242	50.0386093	22.71383485	70.33700415	65.5668928	83.0050957	-1.126221163	0.003766789	GASAL1
ENSG00000164087	162.3745329	157.4131251	151.4255656	341.1344701	385.6876047	302.018541	-1.126544343	7.89E-13	POC1A
ENSG00000143248	13552.18445	13213.32027	12995.55836	28488.24511	29891.75359	28568.75384	-1.12872214	5.49E-266	RGS5
ENSG00000100629	114.6770138	109.4594578	88.69291701	213.6486501	259.3749142	212.0130155	-1.129049482	7.85E-09	CEP128
ENSG00000181938	234.4282318	223.0887998	206.587736	455.4321019	516.8213903	483.0296533	-1.130539213	1.04E-18	GINS3
ENSG00000112118	1119.369436	1237.413109	1158.405577	2555.870888	2612.069303	2563.157353	-1.137148796	2.99E-81	MCM3
ENSG00000169851	469.8713045	454.5173678	417.5019167	967.1338071	1024.96481	961.0589996	-1.137248546	4.26E-36	PCDH7
ENSG00000171345	696.1808097	762.0463208	726.842715	1647.644322	1607.353093	1553.095345	-1.138300863	6.35E-56	KRT19
ENSG00000198554	310.5412941	306.486482	345.0339674	684.0273654	685.5597174	755.0463524	-1.143143668	1.94E-26	WDHD1
ENSG00000229855	214.1314152	202.2393793	231.4647932	457.190527	454.1471546	520.0319248	-1.143536295	2.50E-18	AC008568.1
ENSG00000133119	292.2741592	266.8725829	282.3005188	600.5021729	662.4184611	597.0366522	-1.144017756	4.34E-24	RFC3
ENSG00000114346	599.7709308	568.1467098	562.4378152	1155.285293	1336.40755	1348.082759	-1.148827493	6.50E-40	ECT2
ENSG00000132423	52.77172318	50.0386093	47.59089206	139.7947957	99.31455822	94.00577103	-1.149377868	7.83E-05	COQ3
ENSG00000184344	28.41554325	33.35907287	37.85639141	78.24991712	78.10173996	66.004052	-1.160980867	0.000767358	GDF3
ENSG00000166394	33.48974741	61.5057906	59.48861507	131.0026702	78.10173996	136.0083496	-1.162206081	0.000411473	CYB5R2
ENSG00000175305	92.35051557	80.27026909	99.50822885	183.7554233	256.4822571	169.0103756	-1.163047433	2.30E-07	CCNE2
ENSG00000142856	93.3653564	93.82239244	90.85533938	192.5475489	218.8777157	212.0130155	-1.163955702	1.86E-09	ITGB3BP
ENSG00000167767	314.6006574	288.7644745	341.7891339	676.1144524	744.3770771	708.0434669	-1.171419711	3.41E-27	KRT80
ENSG00000140525	480.0197128	526.4478687	533.2343133	1120.116791	1190.81048	1169.07177	-1.176687949	2.48E-44	FANCI
ENSG00000147642	28.41554325	45.86872519	40.01961377	81.76676732	101.2429962	77.00472733	-1.187239769	0.000340818	SYBU
ENSG00000137135	38.56395156	44.82625417	62.73344862	119.5729071	120.5273765	92.00564824	-1.188594463	6.11E-05	ARHGEF39
ENSG00000140534	144.1073979	156.3706541	165.486511	332.3423446	368.3316625	362.0222246	-1.189627163	1.46E-15	TICRR
ENSG00000163507	350.1200865	300.2316558	346.1155786	744.6930314	776.1963045	756.0464138	-1.192071874	1.34E-30	CIP2A
ENSG00000101945	131.929308	151.1582989	142.7726762	307.7243932	334.5839971	331.0203214	-1.1923977	1.40E-14	SUV39H1
ENSG00000147536	216.1610969	213.7065606	194.690013	423.78045	549.6048367	458.0281184	-1.195257564	1.85E-17	GINS4
ENSG00000138794	268.9328201	274.1698801	271.484407	608.4150859	627.7065767	630.0386782	-1.195722614	9.00E-28	CASP6
ENSG00000144395	41.60847405	50.0386093	38.93800259	99.35101836	107.9925293	92.00564824	-1.196561046	4.37E-05	CCDC150
ENSG00000197467	1383.228052	1287.451719	1287.117308	3111.533221	3026.683478	2938.180375	-1.197359295	9.01E-99	COL13A1
ENSG00000181019	1072.686758	1190.501913	986.429399	2438.056406	2532.039125	2487.152687	-1.197861398	1.94E-69	NQO1
ENSG00000159055	113.662173	106.3320448	124.3852861	283.1064417	253.5896001	254.0155941	-1.200640267	5.06E-12	MIS18A
ENSG00000173597	3049.596696	2965.830072	3084.755094	6898.301682	6916.342972	7192.441545	-1.206831164	1.29E-187	SULT1B1
ENSG00000082153	3734.614256	3821.698785	3889.473814	8494.951676	9202.506249	8729.535908	-1.20719533	1.42E-181	BZW1
ENSG00000105851	124.8254221	135.5212335	110.3243407	271.6766785	292.1583606	299.0183568	-1.217493281	3.58E-13	PIK3CG
ENSG00000092470	223.2649827	174.0926615	229.3015708	452.7944642	547.6763987	459.0281798	-1.21968179	1.94E-17	WDR76
ENSG00000228716	484.0790761	496.2162089	525.663035	1207.158834	1242.878306	1059.065016	-1.221351941	1.36E-41	DHFR
ENSG00000080839	237.4727543	241.8532783	213.0774031	518.7354056	526.4635805	575.0353015	-1.225253903	1.47E-23	RBL1
ENSG00000167670	412.0253772	453.4748968	432.6444732	1039.229236	1036.535438	958.0588154	-1.225327414	8.71E-42	CHAF1A

ENSG00000154920	42.62331488	40.65637006	49.75411442	110.7807815	109.9209673	90.00552545	-1.226036064	2.22E-05	EME1
ENSG00000101000	2488.389716	2571.776024	2489.868944	5652.457496	6228.854816	5782.35498	-1.226069564	3.02E-142	PROCR
ENSG00000164867	1620.700806	1608.532795	1731.659504	3959.973334	3915.693407	3758.230718	-1.230200092	4.26E-123	NOS3
ENSG00000169684	27.40070242	52.12355136	47.59089206	100.2302309	107.9925293	92.00564824	-1.242747645	8.25E-05	CHRNA5
ENSG00000149503	771.2790312	784.9806834	785.2497189	1849.863209	1907.225205	1798.110386	-1.24658637	4.62E-77	INCENP
ENSG00000111206	917.4161108	879.8455469	866.3705577	2082.854535	2221.560603	2026.124384	-1.248666752	1.43E-78	FOXM1
ENSG00000235387	263.8586159	253.3204596	230.383182	535.4404441	610.3506345	634.0389238	-1.249695146	1.16E-24	SPAAR
ENSG00000140379	19.28197578	12.50965233	31.36672431	51.87354056	52.06782664	46.00282412	-1.251754175	0.007177204	BCL2A1
ENSG00000164104	1120.384277	1098.764463	1124.87563	2668.410095	2724.882927	2590.15901	-1.255566234	1.87E-105	HMGB2
ENSG00000146918	599.7709308	503.5135061	548.3768698	1367.175518	1231.307678	1402.086074	-1.275926698	2.22E-47	NCAPG2
ENSG00000088305	105.5434464	166.7953643	140.6094538	369.2692718	330.7271211	299.0183568	-1.276657869	1.09E-12	DNMT3B
ENSG00000171757	18.26713495	20.84942054	20.55061248	56.26960332	54.96048367	33.002026	-1.276699805	0.005771555	LRRC34
ENSG00000166508	1404.539709	1471.96909	1570.499438	3515.970995	3738.277109	3518.215984	-1.276864896	1.08E-112	MCM7
ENSG00000172927	33.48974741	28.14671773	49.75411442	96.71338071	81.958616	91.00558685	-1.279108159	9.57E-05	MYEOV
ENSG00000125885	216.1610969	240.8108073	194.690013	508.184855	547.6763987	527.0323546	-1.279362752	4.07E-24	MCM8
ENSG00000167513	333.8826332	399.2664034	402.3593601	890.642315	973.8612019	891.054702	-1.279799969	9.25E-37	CDT1
ENSG00000075702	187.7455536	202.2393793	191.4451749	480.0500533	454.1471546	478.0293463	-1.280498348	1.65E-23	WDR62
ENSG00000171476	52.77172318	29.18918876	27.04027958	83.52519243	76.17330193	106.0065078	-1.280742396	0.000285948	HOPX
ENSG00000164109	281.11091	298.1467138	299.6062977	675.2352398	742.4486391	727.0446334	-1.286909215	8.77E-34	MAD2L1
ENSG00000149781	1033.107965	1134.208478	1117.304352	2751.935287	2688.242605	2596.159379	-1.291440119	1.82E-100	FERMT3
ENSG00000101842	36.5342699	18.76447849	31.36672431	67.69936649	60.74579774	84.00515709	-1.291867107	0.00071447	VSIG1
ENSG00000113810	1404.539709	1205.096507	1268.729918	3185.387075	3274.487764	3058.187743	-1.294928108	5.32E-96	SMC4
ENSG00000186638	54.80140485	78.18532704	61.65183744	152.1037715	136.9190997	189.0116035	-1.295763956	1.47E-07	KIF24
ENSG00000010292	1067.612554	1123.783767	1117.304352	2646.429781	2800.09201	2678.164413	-1.296140851	2.64E-108	NCAPD2
ENSG00000140465	184.7010311	143.8610017	135.2013979	379.8198224	417.5068321	348.0213651	-1.302862208	2.68E-16	CYP1A1
ENSG00000203760	133.9589896	167.8378354	135.2013979	375.4237597	361.5821294	344.0211195	-1.307122939	1.89E-17	CENPW
ENSG00000228649	22.32649827	34.4015439	55.16217034	90.55889284	86.77971106	99.006078	-1.308899275	0.000182116	SNHG26
ENSG00000186193	276.0367059	310.6563661	279.0556852	771.069408	719.3073828	655.040213	-1.310101468	1.64E-31	SAPCD2
ENSG00000185347	105.5434464	157.4131251	184.9555123	390.370373	368.3316625	355.0217948	-1.316991132	1.20E-13	TEDC1
ENSG00000285108	19.28197578	21.89189157	25.95866839	77.37070456	39.53297948	50.0030697	-1.319203104	0.003017558	AC103718.1
ENSG00000094804	329.8232699	312.7413081	373.1558582	835.2519243	841.7631973	857.0526146	-1.319647282	2.21E-39	CDC6
ENSG00000162073	133.9589896	151.1582989	146.0175097	392.1287981	363.5105675	325.019953	-1.327268954	6.24E-18	PAQR4
ENSG00000112029	199.9236436	169.9227774	233.6280156	494.9966667	494.6443531	525.0322318	-1.328459527	1.31E-22	FBXO5
ENSG00000076003	696.1808097	727.6447769	723.5978815	1721.498177	2010.39664	1669.102466	-1.330701512	3.06E-60	MCM6
ENSG00000137310	511.4797786	459.729723	498.6227554	1184.299307	1316.158951	1211.074348	-1.336016676	1.67E-54	TCF19
ENSG00000129173	394.7730831	433.6679473	432.6444732	1025.161835	1091.495921	1074.065937	-1.33940081	2.36E-51	E2F8
ENSG00000120802	1203.601225	1301.003842	1188.69069	3022.732753	3313.056525	3011.184857	-1.339407745	3.36E-105	TMPO
ENSG00000169247	13.1929308	20.84942054	16.22416775	51.87354056	40.4971985	35.00214879	-1.344973964	0.006131412	SH3TC2
ENSG00000180385	37.54911073	42.74131211	40.01961377	88.80046774	103.1714343	114.0069989	-1.345357915	4.56E-06	EMC3-AS1
ENSG00000136928	720.5369896	766.2162049	701.9656578	1754.908254	1887.940825	1927.118306	-1.346906086	2.72E-78	GABBR2
ENSG00000128408	19.28197578	19.80694952	21.63222366	50.11511546	43.38985553	61.00374503	-1.347248075	0.001726148	RIBC2
ENSG00000177602	75.09822146	94.86486347	96.2633953	232.9913262	221.7703727	224.0137522	-1.352063683	2.17E-12	HASPIN
ENSG00000187741	296.3335225	344.015439	325.5649661	866.9035761	757.8761433	840.0515709	-1.352328727	1.14E-37	FANCA
ENSG00000100479	96.4098789	84.4401532	87.61050583	215.4070752	216.9492777	255.0156555	-1.354854209	1.79E-12	POLE2
ENSG00000121621	226.3095052	189.7297269	219.5670702	542.4741445	583.3525021	500.030697	-1.355167544	4.56E-26	KIF18A
ENSG00000139618	181.6565087	175.1351326	169.8129557	439.6062759	431.0058983	479.0294077	-1.356901641	4.01E-24	BRCA2
ENSG00000166845	179.626827	188.6872559	169.8129557	429.9349379	498.5012291	451.0276887	-1.357203048	1.13E-23	C18orf54
ENSG00000167900	496.2571661	516.0231584	607.8654849	1258.153162	1543.714638	1350.082882	-1.358146981	2.64E-44	TK1
ENSG00000147697	60.89044983	52.12355136	64.89667099	166.1711723	149.4539468	142.0087179	-1.364694738	7.86E-09	GSDMC
ENSG00000175832	187.7455536	208.4942054	197.9348465	503.7887922	563.1039029	463.0284254	-1.364962349	5.45E-25	ETV4
ENSG00000213707	16.23745329	20.84942054	19.4690013	48.35669035	43.38985553	54.00331527	-1.366316806	0.001952212	HMGB1P10
ENSG00000164167	66.97949481	59.42084855	68.14150454	184.6346359	168.7383271	148.0090863	-1.367401333	1.66E-09	LSM6
ENSG00000129514	36.5342699	35.44401492	22.71383485	77.37070456	95.45768217	74.00454315	-1.3791582	5.89E-05	FOXA1
ENSG00000012048	279.0812284	281.4671773	245.5257386	683.1481528	709.6651927	717.0440194	-1.387008013	3.13E-37	BRCA1
ENSG00000087586	338.9568374	413.8609978	359.0949128	925.8108171	1022.072153	971.0596135	-1.392258951	1.85E-44	AURKA
ENSG00000100297	885.956045	950.7335767	975.6132872	2403.767117	2570.607886	2424.148819	-1.395856265	1.13E-105	MCM5
ENSG00000135476	312.5709758	335.6756707	341.7891339	938.1197928	861.0475776	814.0499747	-1.401588726	3.61E-43	ESPL1
ENSG00000101695	24.35617993	26.06177568	10.81611183	54.51117822	52.06782664	56.00343806	-1.404274576	0.001507575	RNF125
ENSG00000073111	512.4946194	498.301151	523.4998126	1363.658668	1399.081786	1298.079689	-1.404549888	9.65E-70	MCM2
ENSG00000097046	105.5434464	139.6911176	104.9162848	301.5699053	324.941807	302.018541	-1.406616045	1.65E-16	CDC7
ENSG00000167325	1447.163024	1294.749016	1300.096642	3290.892582	3773.953212	3665.225009	-1.407749156	6.61E-105	RRM1
ENSG00000118276	232.3985502	214.7490316	206.587736	597.8645353	564.0681219	575.0353015	-1.409345236	1.59E-32	B4GALT6
ENSG00000212232	21.31165744	17.72200746	9.734500648	55.39039077	44.35407454	30.00184182	-1.410513505	0.006398276	SNORD17
ENSG00000214826	102.4989239	102.1621607	148.1807321	336.7384074	247.804286	353.0216721	-1.412025392	7.43E-13	DDX12P
ENSG00000206432	14.20777163	23.97683362	38.93800259	62.42409118	61.71001676	81.00497291	-1.415731239	0.000630811	TMEM200C
ENSG00000129810	74.08338062	80.27026909	71.38633808	184.6346359	219.8419347	199.0122174	-1.417647803	4.79E-12	SGO1
ENSG00000171320	138.0183529	115.714284	124.3852861	296.29463	341.3335302	376.0230841	-1.420728901	8.39E-18	ESCO2
ENSG00000013810	1018.900194	947.6061637	1080.529572	2729.954974	2797.199353	2645.162387	-1.423788201	8.35E-114	TACC3
ENSG00000226167	16.23745329	9.382239244	17.30577893	33.41007697	29.89078937	52.00319248	-1.42404709	0.008519324	AP4B1-AS1
ENSG00000101412	274.0070242	240.8108073	244.4441274	654.1341386	743.4128581	644.0395377	-1.4262411	9.66E-35	E2F1
ENSG00000089685	470.8861453	483.7065566	554.8665369	1311.785127	1334.479112	1414.086811	-1.428188828	4.73E-63	BIRC5
ENSG00000111788	38.56395156	47.95366725	23.79544603	128.3650326	83.88705403	85.00521848	-1.430074807	2.98E-05	AC009533.1
ENSG00000123219	147.1519204	136.5637046	188.2003459	431.693363	422.3279272	416.0255399	-1.430182693	5.21E-22	CENPK
ENSG00000117593	377.5207889	410.7335847	400.1961377	1032.195536	1097.281235	1093.067104	-1.439059113	9.17E-59	DARS2
ENSG00000085840	135.9886713	170.9652484	118.9772301	368.3900592	349.0472823	439.0269519	-1.439519849	5.10E-18	ORC1
ENSG00000104738	1340.604737	1318.725849	1263.321862	3391.122813	3733.456014	3549.217887	-1.443618713	3.22E-142	MCM4
ENSG00000137807	470.8861453	448.2625417	471.5824758	1174.627969	1298.803009	1327.08147	-1.449706493	8.93E-64	KIF23
ENSG00000127423	38.56395156	29.18918876	35.69316904	101.1094435	95.45768217	87.00534127	-1.455376368	2.23E-06	AUNIP
ENSG00000113368	1129.517844	1160.270253	1174.629745	3069.331019	3251.346508	3203.196645	-1.458895659	1.85E-150	LMNB1
ENSG00000116574	36.5342699	17.72200746	20.55061248	72.09542925	67.49533083	67.00411339	-1.462280855	0.000173411	RHOU
ENSG00000176208	96.4098789	105.2895737	135.2013979	325.3086442	298.9078937	305.0187251	-1.466389694	2.04E-17	ATAD5
ENSG00000163584	423.1886263	429.4980632	365.5845799	1075.276951	1202.381108	1121.068823	-1.478974496	1.90E-56	RPL22L1
ENSG00000111665	194.8494395	142.8185307	165.486511	465.9826525	464.7535637	473.0290393	-1.479228534	7.26E-26	CDCA3
ENSG00000136982	101.484083	83.39768217	95.18178411	218.9239254	283.4803895	280.0171903	-1.480385121	9.94E-15	DSCC1
ENSG00000011426	1314.218875	1186.332029	1237.363193	3260.120142	3661.139588	3517.215922	-1.481033976	9.94E-129	ANLN
ENSG00000100162	56.83108651	70.88802985	74.63117163	198.7020367	197.6648974	169.0103756	-1.484884334	9.85E-12	CENPM
ENSG00000163535	279.0812284	291.8918876	294.1982418	755.2435821	847.5485114	823.0505272	-1.487149759	1.51E-46	SGO2
ENSG00000166803	97.42471973	97.99227655	122.2220637	309.4828183	288.3014845	292.017927	-1.488159553	3.47E-18	PCLAF
ENSG00000122952	392.7434014	432.6254763	577.5803718	1312.66434	1425.115699	1203.073857	-1.49064544	1.21E-14	ZWINT
ENSG00000215784	23.3413391	20.84942054	19.4690013	75.61227946	48.21095059	55.00337667	-1.491511116	0.000282495	FAM72D
ENSG00000138182	344.0310415	342.9729679	296.3614642	851.9569628	959.3979168	958.0588154	-1.492218144	4.94E-48	KIF20B
ENSG00000068489	590.6373633	548.3397603	526.7446462	1503.453464	1697.025461	1493.091661	-1.493808419	5.47E-73	PRR11
ENSG00000256663	42.62331488	59.42084855	52.99894797	139.7947957	149.4539468	148.0090863	-1.496601542	1.26E-09	AC112777.1

ENSG00000161800	315.6154983	373.2046277	377.4823029	951.3079811	1029.785905	1032.063359	-1.499169635	1.39E-52	RACGAP1
ENSG00000111247	159.3300104	142.8185307	148.1807321	416.7467496	443.5407454	418.0256627	-1.504605138	3.61E-27	RAD51AP1
ENSG00000140451	150.1964429	167.8378354	173.0577893	505.5472173	470.5388778	425.0260924	-1.514333451	1.55E-27	PIF1
ENSG00000242193	145.1222388	127.1814653	111.4059519	389.4911605	336.5124351	375.0230227	-1.519508551	8.88E-22	CRYZL2P
ENSG00000090889	415.0698997	353.3976782	338.5443003	1030.437111	1111.744521	1048.064341	-1.52566455	9.67E-56	KIF4A
ENSG00000146410	38.56395156	37.52895698	34.61155786	105.5055062	108.9567483	105.0064464	-1.528137272	8.44E-08	MTFR2
ENSG00000100558	51.75688235	57.33590649	86.52889465	183.7554233	194.7722404	185.0113579	-1.529745524	3.74E-11	PLEK2
ENSG00000249992	40.59363322	62.54826163	83.2840611	181.9969982	181.2731742	174.0106825	-1.531211377	6.96E-10	TMEM158
ENSG00000123080	131.929308	159.4980672	139.5278426	424.6596626	448.3618405	384.0235753	-1.545043275	9.55E-26	CDKN2C
ENSG00000178401	14.20777163	15.63706541	14.06094538	38.68535228	43.38985553	47.00288551	-1.554293765	0.00109958	DNAJC22
ENSG00000165480	190.7900761	187.6447849	177.384234	524.0106809	615.1717295	497.0305128	-1.557194884	1.11E-31	SKA3
ENSG00000122966	294.3038408	296.0617717	250.9337945	824.7013737	844.6558544	820.050343	-1.564040667	4.53E-51	CIT
ENSG00000101447	327.7935882	371.1196857	348.278801	932.8445175	1101.138111	1073.065876	-1.568532934	4.59E-55	FAM83D
ENSG00000101003	237.4727543	211.6216185	223.8935149	686.665003	672.0606512	638.0391693	-1.569076315	2.46E-43	GINS1
ENSG00000265415	72.05369896	70.88802985	71.38633808	215.4070752	208.2713066	219.0134453	-1.584213193	1.11E-15	AC099850.3
ENSG00000198056	55.81624568	33.35907287	61.65183744	137.1571581	157.1676989	158.0097002	-1.584729919	1.64E-09	PRIM1
ENSG00000102384	129.8996263	120.9266391	123.3036749	320.9125814	382.7949477	420.0257854	-1.58492411	8.56E-23	CENPI
ENSG00000143476	202.9681661	201.1969082	186.0371235	587.3139847	588.1735972	600.0368364	-1.58821609	8.93E-41	DTL
ENSG00000181544	26.38586159	28.14671773	47.59089206	108.1431439	94.49346316	107.0065692	-1.60480527	3.82E-07	FANCB
ENSG00000079616	420.1441038	413.8609978	385.0535812	1246.723399	1268.91222	1202.073796	-1.608203952	7.80E-80	KIF22
ENSG00000153044	58.86076817	75.05791395	61.65183744	169.6880225	228.5199058	201.0123402	-1.614318551	3.27E-13	CENPH
ENSG00000276043	655.5871765	651.544392	639.2322092	1968.556904	2015.217735	1999.122726	-1.619782113	8.90E-129	UHRF1
ENSG00000104147	50.74204152	43.78378314	57.3253927	150.3453464	178.3805172	138.0084724	-1.621084166	5.09E-11	OIP5
ENSG00000088325	1097.042938	1123.783767	1145.426243	3458.822179	3508.792984	3437.211011	-1.628303281	4.48E-203	TPX2
ENSG00000076382	298.3632042	334.6331997	334.2178556	1012.85286	964.2190118	1023.062806	-1.633844628	7.46E-66	SPAG5
ENSG00000185432	268.9328201	251.2355175	218.485459	794.8081469	831.1567882	669.0410725	-1.634931736	1.99E-41	METTL7A
ENSG00000084734	39.57879239	69.84555882	48.67250324	189.9099112	155.2392609	146.0089635	-1.637562954	2.29E-10	GCKR
ENSG00000187951	38.56395156	51.08108033	47.59089206	135.398733	168.7383271	127.007797	-1.652650465	2.23E-10	AC091057.1
ENSG00000148773	1444.118502	1243.667935	1254.686972	4350.343707	3753.704613	4318.265099	-1.655417695	3.05E-128	MKI67
ENSG00000144354	298.3632042	257.4903437	290.9534083	867.7827887	943.9704126	860.0527988	-1.657543606	6.27E-58	CDCA7
ENSG00000284946	18.26713495	30.23165979	40.01961377	74.73306691	105.0998723	99.006078	-1.658379274	2.10E-06	AC068831.7
ENSG00000109674	45.66783737	72.9729719	42.18283614	158.2582593	188.0227073	162.0099458	-1.659063031	4.55E-11	NEIL3
ENSG00000118193	187.7455536	213.7065606	190.3635682	595.2268976	603.6011014	672.0412567	-1.659877242	3.92E-42	KIF14
ENSG00000196584	92.35051557	80.27026909	87.61050583	287.5025045	265.1602282	278.0170675	-1.674512544	3.55E-21	XRCC2
ENSG00000160298	76.11306229	85.48262422	90.85533938	285.7440794	229.4841248	293.0179884	-1.680137456	9.37E-19	C21orf58
ENSG00000171241	368.3872215	327.3359025	373.1558582	1091.981989	1213.951736	1134.069621	-1.68616796	2.41E-73	SHCBP1
ENSG00000108342	23.3413391	23.97683362	19.4690013	83.52519243	71.35220687	60.00368364	-1.686584598	5.46E-06	CSF3
ENSG00000136492	77.12790312	101.1196896	97.34500648	274.3143162	314.3353979	300.0184182	-1.689868714	2.56E-21	BRIP1
ENSG00000189184	20.29861661	14.59459438	9.734500648	43.96062759	60.74579774	40.00245576	-1.692212011	0.000437776	PCDH18
ENSG00000165304	330.8381107	361.7374464	336.3810779	1099.01569	1164.776566	1063.065262	-1.693090055	1.24E-75	MELK
ENSG00000101057	700.240173	795.4053937	726.842715	2385.303653	2431.760348	2385.146425	-1.696383843	5.41E-145	MYBL2
ENSG00000146670	380.5653114	394.0540483	336.3810779	1146.493168	1298.803009	1162.07134	-1.698100091	2.58E-72	CDCA5
ENSG00000117724	641.3794049	600.4633116	630.5793197	2161.104453	1896.618796	2030.12463	-1.701363228	2.84E-116	CENPF
ENSG00000164619	8.118726644	12.50965233	10.81611183	35.16850207	50.13938861	17.0010437	-1.705215518	0.006009485	BMPER
ENSG00000071539	304.4522491	380.5019249	375.3190805	1127.150492	1221.665488	1110.068147	-1.706914953	3.39E-67	TRIP13
ENSG00000072571	235.4430727	239.7683562	293.1166306	842.2856247	840.7989783	823.0505272	-1.707298138	1.21E-55	HMMR
ENSG00000142945	270.9625017	260.6177568	292.0350194	829.976649	942.0419745	920.0564824	-1.708594126	1.76E-60	KIF2C
ENSG00000168078	163.3893737	183.4749008	218.485459	612.8111487	631.5634527	607.0372661	-1.7133407	1.04E-41	PBK
ENSG00000198826	457.6932145	379.4594539	398.0329154	1383.001344	1353.763493	1336.082022	-1.720659035	6.12E-87	ARHGAP11A
ENSG00000129195	134.9738305	120.9266391	150.3439545	464.2242274	475.3599728	405.0248645	-1.728220596	9.19E-32	PIMREG
ENSG00000142731	207.0275294	172.0077195	193.6084018	643.583588	646.0267379	617.0378801	-1.7351466	2.90E-47	PLK4
ENSG00000184661	185.715872	232.4710391	168.7313446	596.9853227	672.0606512	687.0421776	-1.735367095	6.30E-40	CDCA2
ENSG00000174371	90.32083391	80.27026909	120.0588413	297.1738425	347.1188443	323.0198302	-1.736285923	5.45E-22	EXO1
ENSG00000133110	156.2854879	177.2200746	167.6497334	544.2325696	634.4561098	492.0302058	-1.737674111	4.12E-36	POSTN
ENSG00000051341	97.42471973	95.9073345	80.03922755	307.7243932	295.0510176	313.0192163	-1.742689837	3.18E-24	POLQ
ENSG00000134057	617.0232249	691.158291	697.6392131	2130.332013	2445.259414	2141.131444	-1.743990121	4.43E-113	CCNB1
ENSG00000189057	282.1257509	295.0193007	260.6682951	907.3473535	978.682297	935.0574033	-1.75070045	4.57E-69	FAM111B
ENSG00000131153	169.4784187	157.4131251	125.4668972	466.861865	559.2470269	499.0306356	-1.751023871	1.45E-33	GINS2
ENSG00000156509	16.23745329	9.382239244	15.14255656	47.4774778	30.85500838	59.00362224	-1.751550532	0.000516022	FBXO43
ENSG00000100526	104.5286055	128.2239363	120.0588413	375.4237597	387.6160428	426.0261538	-1.75301127	4.33E-30	CDKN3
ENSG00000134690	250.6656851	302.3165979	270.4027958	920.5355418	979.646516	891.054702	-1.761680721	1.11E-64	CDCA8
ENSG00000166250	163.3893737	169.9227774	164.4048998	560.0583955	552.4974938	578.0354857	-1.764001038	1.09E-45	CLMP
ENSG00000143228	155.2706471	145.9459438	178.4658452	473.0163529	589.1378162	577.0354243	-1.772767759	3.15E-36	NUF2
ENSG00000169607	131.929308	168.8803064	188.2003459	561.8168206	593.9589113	513.0314951	-1.773242279	3.34E-36	CKAP2L
ENSG00000166851	585.5631592	618.1853191	597.0493731	2033.618632	2126.102921	2002.122911	-1.774824768	2.51E-144	PLK1
ENSG00000138160	434.3518754	375.2895698	447.7870298	1437.512522	1481.040402	1423.087364	-1.788076753	1.20E-98	KIF11
ENSG00000085999	56.83108651	47.95366725	77.87600518	230.3536886	210.1997446	189.0116035	-1.788730646	1.66E-15	RAD54L
ENSG00000241472	11.16324914	10.42471027	4.326444732	32.53086442	32.7834464	25.00153485	-1.796466828	0.003883894	PTPRG-AS1
ENSG00000110675	832.169481	803.7451619	820.942888	2816.997016	3006.434879	2750.168833	-1.803030589	2.79E-182	ELMOD1
ENSG00000139549	39.57879239	19.80694952	30.28511313	94.07574305	91.60080612	128.0078584	-1.803526909	4.95E-08	DHH
ENSG00000117399	760.115782	781.8532704	946.4097852	2974.376063	3009.327536	2724.167237	-1.808314332	6.25E-126	CDC20
ENSG00000213347	64.94981315	79.22779806	81.12083873	314.7580936	242.018972	233.0143048	-1.812707295	3.38E-19	MXD3
ENSG00000112742	158.3151696	148.0308859	143.8542874	495.8758793	544.7837417	543.0333369	-1.813363443	5.08E-43	TTK
ENSG00000240583	137.0035121	144.9034728	140.6094538	472.1371404	598.7800063	415.0254785	-1.814539629	1.15E-30	AQP1
ENSG00000093009	171.5081004	176.1776036	181.7106788	606.6566608	615.1717295	645.0395991	-1.818289933	6.95E-52	CDC45
ENSG00000144554	130.9144671	135.5212335	105.9978959	444.0023387	451.2544975	420.0257854	-1.819021796	6.55E-35	FANCD2
ENSG00000112984	469.8713045	504.5559771	423.9915838	1550.930942	1840.694094	1548.095038	-1.819900348	1.95E-87	KIF20A
ENSG00000284906	13.1929308	14.59459438	12.9793342	65.06172884	31.81922739	47.00288551	-1.822069447	0.000217439	AC091057.6
ENSG00000137804	44.65299654	43.78378314	34.61155786	132.7610953	147.5255088	157.0096388	-1.826442738	1.03E-12	NUSAP1
ENSG00000170312	287.199955	283.5521194	255.2602392	892.4007402	1081.853731	958.0588154	-1.826558192	5.26E-65	CDK1
ENSG00000186871	85.24662976	97.99227655	73.54956045	288.381717	308.5500838	317.0194619	-1.829806944	4.87E-25	ERCC6L
ENSG00000007968	34.50458824	37.52895698	49.75411442	147.7077087	140.7759757	144.0088407	-1.831416329	1.35E-12	E2F2
ENSG00000138587	26.38586159	13.55212335	23.79544603	51.87354056	82.92283502	93.00570964	-1.834401808	4.80E-06	MNS1
ENSG00000165071	38.56395156	31.27413081	36.77478023	88.80046774	167.7741081	126.0077356	-1.841251387	2.87E-09	TMEM71
ENSG00000135547	37.54911073	31.27413081	55.16217034	147.7077087	152.3466039	146.0089635	-1.850384021	2.54E-12	HEY2
ENSG00000146678	14.20777163	9.382239244	9.734500648	35.16850207	43.38985553	42.00257854	-1.850932195	0.000335234	IGFBP1
ENSG00000154839	153.2409654	160.5405382	177.384234	579.4010717	621.9212626	582.0357313	-1.861284644	1.03E-49	SKA1
ENSG00000105011	166.4338962	183.4749008	221.7302925	673.4768147	768.4825524	636.0390465	-1.863863549	1.89E-46	ASF1B
ENSG00000075218	293.289	359.6525044	350.4420233	1211.554896	1166.705004	1274.078216	-1.864800145	2.76E-85	GTSE1
ENSG00000197299	82.20210727	71.93050087	84.36567228	276.9519538	289.2657035	308.0189093	-1.873743307	1.06E-25	BLM
ENSG00000151725	159.3300104	121.9691102	146.0175097	496.7550918	561.1754649	511.0313723	-1.875644841	5.34E-41	CENPU
ENSG00000041353	6.089044983	16.67953643	11.89772301	47.4774778	48.21095059	32.00196461	-1.885799197	0.000389578	RAB27B
ENSG00000024526	200.9384844	232.4710391	204.4245136	742.9346063	816.693503	804.0493607	-1.889295013	1.00E-63	DEPDC1

ENSG00000121152	179.626827	178.2625456	139.5278426	605.7774482	641.2056429	606.0372047	-1.8954937	2.56E-49	NCAPH
ENSG00000092853	181.6565087	241.8532783	191.4451794	722.7127176	795.4806847	771.0473347	-1.896065328	2.52E-55	CLSPN
ENSG00000183856	243.5617993	251.2355175	244.4441274	995.2686087	894.795243	872.0535355	-1.902419363	9.33E-74	IQGAP3
ENSG00000127564	158.3151696	137.6061756	147.0991209	554.7831202	544.7837417	559.0343192	-1.904016388	4.07E-49	PKMYT1
ENSG00000109805	399.8472872	407.6061716	401.2777489	1445.425435	1586.140274	1536.094301	-1.917578025	6.34E-122	NCAPG
ENSG00000134222	56.83108651	71.93050087	99.50822885	291.8985672	314.3353979	255.0156555	-1.919705245	6.38E-21	PSRC1
ENSG00000157456	228.3391869	248.1081045	213.0774031	809.7547603	989.2887061	825.05065	-1.927343967	1.91E-61	CCNB2
ENSG00000196550	27.40070242	27.10424671	23.79544603	116.0560568	96.42190118	86.00527988	-1.931311972	1.54E-09	FAM72A
ENSG00000186185	258.7844118	256.4478727	271.484407	1032.195536	979.646516	995.061087	-1.935016668	2.55E-88	KIF18B
ENSG00000123485	183.6861903	181.3899587	203.3429024	727.1087804	746.3055151	700.0429757	-1.935936073	4.84E-64	HJURP
ENSG00000171848	421.1589446	433.6679473	412.0938608	1668.745423	1644.957634	1560.095775	-1.943859504	1.05E-134	RRM2
ENSG00000188610	38.56395156	30.23165979	33.52994668	140.6740083	134.9906617	119.0073059	-1.947401602	1.06E-12	FAM72B
ENSG00000091651	103.5137647	140.7335887	128.7117308	483.5669035	513.9287333	452.0277501	-1.959967126	4.77E-40	ORC6
ENSG00000169679	320.6897024	348.1853231	316.9120766	1192.21222	1343.157083	1306.08018	-1.961631008	1.96E-101	BUB1
ENSG00000152253	34.50458824	72.9729719	46.50928087	232.9913262	179.3447362	187.0114807	-1.962726052	2.08E-14	SPC25
ENSG00000163808	137.0035121	114.671813	117.895619	448.3984015	475.3599728	523.032109	-1.96708437	8.36E-42	KIF15
ENSG00000156970	271.9773426	227.2586839	228.2199596	961.8585317	1024.96481	878.0539039	-1.976682089	9.25E-73	BUB1B
ENSG00000135451	142.0777163	150.1158279	178.4658452	640.9459503	638.3129858	573.0351787	-1.978749113	4.63E-52	TROAP
ENSG00000145386	378.5356298	478.4942015	465.0928087	1694.242587	1887.940825	1652.101423	-1.986092034	4.88E-106	CCNA2
ENSG00000237649	255.7398893	265.8301119	288.7901859	1085.827502	1019.179495	1126.06913	-1.996200649	3.70E-92	KIFC1
ENSG00000182010	9.133567474	9.382239244	16.22416775	48.35669035	33.74766541	57.00349945	-2.005965865	7.52E-05	RTKN2
ENSG00000133101	50.74204152	78.18532704	67.05989335	269.9182534	269.9813233	251.0154099	-2.01471853	5.50E-24	CCNA1
ENSG00000065328	156.2854879	136.5637046	148.1807321	618.086424	646.0267379	536.0329071	-2.029377669	5.66E-52	MCM10
ENSG00000261253	5.074204152	5.212355136	6.489667099	34.28928952	13.49906617	21.00128927	-2.041910033	0.007243622	AC137932.2
ENSG00000132470	63.93497232	64.63320368	46.50928087	237.387389	284.4446085	207.0127085	-2.055649493	3.50E-21	ITGB4
ENSG00000178999	172.5229412	187.6447849	216.3222366	827.3390113	730.878011	836.0513253	-2.055940048	7.27E-67	AURKB
ENSG00000138180	426.2331488	394.0540483	465.0928087	1729.41109	1883.11973	1732.106334	-2.056468113	4.70E-139	CEP55
ENSG00000137812	319.6748616	286.6795325	247.6889609	1156.164506	1156.098595	1255.077049	-2.060475827	6.57E-94	KNL1
ENSG00000175063	210.0720519	233.5135101	235.7912379	879.2125519	973.8612019	993.0609642	-2.066852508	5.31E-84	UBE2C
ENSG00000080986	176.5823045	134.4787625	184.9555123	689.3026407	712.5578497	741.0454929	-2.111340651	2.65E-62	NDC80
ENSG00000115163	64.94981315	79.22779806	57.3253927	283.9856543	312.4069598	288.0176815	-2.132589516	9.37E-30	CENPA
ENSG00000185862	7.103885813	6.254826163	11.89772301	23.7387389	46.28251257	41.00251715	-2.137821646	0.000281166	EVI2B
ENSG00000158402	43.63815571	38.571428	36.77478023	175.8425104	158.1319179	200.0122788	-2.164169137	6.52E-19	CDC25C
ENSG00000126787	447.5448062	457.6447809	432.6444732	1935.146827	2144.423082	2028.124507	-2.190154394	2.79E-182	DLGAP5
ENSG00000131747	973.2323564	888.1853151	996.1638996	4205.273636	4578.111868	4267.261968	-2.191338261	1.14E-298	TOP2A
ENSG00000161888	72.05369896	77.14285601	88.69211701	351.6850207	360.6179104	386.0236981	-2.208205479	1.86E-39	SPC24
ENSG00000066279	440.4409204	395.0965193	362.3397463	1868.326673	1782.840953	1917.117692	-2.215378063	3.03E-160	ASPM
ENSG00000261008	4.059363322	2.084942054	6.489667099	15.82582593	21.21281826	23.00141206	-2.251918703	0.005858025	LINC01572
ENSG00000239556	5.074204152	3.127413081	3.244833549	21.9803138	22.17703727	11.00067533	-2.267798401	0.008721522	AC004951.2
ENSG00000065618	32.47490658	39.61389903	36.77478023	161.7751095	185.1300503	182.0111737	-2.280617733	1.41E-20	COL17A1
ENSG00000121211	14.20777163	8.339768217	10.81611183	54.51117822	65.5668928	46.00282412	-2.313726794	6.28E-07	MND1
ENSG00000256612	5.074204152	4.169884109	7.571278282	22.85952635	35.67610344	25.00153485	-2.31567195	0.000643805	CYP2B7P
ENSG00000171346	15.22261246	29.18918876	24.87705721	123.9689698	119.5631575	108.0066305	-2.346801956	1.07E-13	KRT15
ENSG00000123689	84.23178893	123.0115812	94.10017293	487.9629663	536.1057706	522.0320476	-2.359252159	1.43E-53	G0S2
ENSG00000102900	316.6303391	345.05791	296.3614642	1633.576921	1660.385138	1647.101116	-2.365988755	9.58E-176	NUP93
ENSG00000163814	16.23745329	15.63706541	28.12189076	121.3313322	107.9925293	91.00558685	-2.423230072	1.15E-12	CDCP1
ENSG00000035499	56.83108651	56.29343547	71.38633808	285.7440794	333.6197781	379.0232683	-2.436558572	2.56E-36	DEPDC1B
ENSG00000179750	5.074204152	14.59459438	14.06094538	61.54487863	63.63845478	63.00386782	-2.486398893	3.80E-08	APOBEC3B
ENSG00000163072	1.01484083	4.169884109	6.489667099	14.94661338	38.56876047	17.0010437	-2.604548365	0.001975128	NOSTRIN
ENSG00000099937	137.0035121	126.1389943	141.691065	1221.226235	1147.420624	1138.069866	-3.115538025	3.08E-175	SERPIND1
ENSG00000187513	7.103885813	17.72200746	5.408055916	236.5081765	296.9794556	204.0125244	-4.605212788	1.76E-40	GJA4
ENSG00000231066	0	0	0	1.758425104	8.677971106	9.000552545	-5.068353394	0.0099406	NPM1P9
ENSG00000224715	0	0	0	7.033700415	5.785314071	7.000429757	-5.102010875	0.006763631	Z82186.1
